# The correlation between CpG methylation and gene expression is driven by sequence variants

**DOI:** 10.1038/s41588-024-01851-2

**Published:** 2024-07-24

**Authors:** Olafur Andri Stefansson, Brynja Dogg Sigurpalsdottir, Solvi Rognvaldsson, Gisli Hreinn Halldorsson, Kristinn Juliusson, Gardar Sveinbjornsson, Bjarni Gunnarsson, Doruk Beyter, Hakon Jonsson, Sigurjon Axel Gudjonsson, Thorunn Asta Olafsdottir, Saedis Saevarsdottir, Magnus Karl Magnusson, Sigrun Helga Lund, Vinicius Tragante, Asmundur Oddsson, Marteinn Thor Hardarson, Hannes Petur Eggertsson, Reynir L. Gudmundsson, Sverrir Sverrisson, Michael L. Frigge, Florian Zink, Hilma Holm, Hreinn Stefansson, Thorunn Rafnar, Ingileif Jonsdottir, Patrick Sulem, Agnar Helgason, Daniel F. Gudbjartsson, Bjarni V. Halldorsson, Unnur Thorsteinsdottir, Kari Stefansson

**Affiliations:** 1 deCODE genetics/Amgen Inc., Reykjavik, Iceland; 2 School of Technology, Reykjavik University, Reykjavik, Iceland; 3 School of Engineering and Natural Sciences, University of Iceland, Reykjavik, Iceland; 4 Faculty of Medicine, School of Health Sciences, University of Iceland, Reykjavik, Iceland; 5 Department of Anthropology, University of Iceland, Reykjavik, Iceland

**Keywords:** DNA sequencing, Epigenetics, Genome-wide association studies, Gene expression, Genomics

## Abstract

Gene promoter and enhancer sequences are bound by transcription factors and are depleted of methylated CpG sites (cytosines preceding guanines in DNA). The absence of methylated CpGs in these sequences typically correlates with increased gene expression, indicating a regulatory role for methylation. We used nanopore sequencing to determine haplotype-specific methylation rates of 15.3 million CpG units in 7,179 whole-blood genomes. We identified 189,178 methylation depleted sequences where three or more proximal CpGs were unmethylated on at least one haplotype. A total of 77,789 methylation depleted sequences (~41%) associated with 80,503 *cis*-acting sequence variants, which we termed allele-specific methylation quantitative trait loci (ASM-QTLs). RNA sequencing of 896 samples from the same blood draws used to perform nanopore sequencing showed that the ASM-QTL, that is, DNA sequence variability, drives most of the correlation found between gene expression and CpG methylation. ASM-QTLs were enriched 40.2-fold (95% confidence interval 32.2, 49.9) among sequence variants associating with hematological traits, demonstrating that ASM-QTLs are important functional units in the noncoding genome.

## Abstract (web summary)

Allele-specific DNA methylation data in whole blood from 7,179 individuals sequenced by Nanopore, and gene expression profiles from 896 samples, show that DNA sequence variability accounts for most of the correlation between CpG methylation and gene expression.

## Main

Cytosines preceding guanines in DNA (CpG dinucleotides) are modified predominantly by the addition of a methyl group in humans and other vertebrates^1^. This modification is commonly known as CpG methylation, or 5-mCpG, where the number 5 indicates the position of the methyl group on the carbon ring of cytosine. DNA methyltransferases (DNMTs) are responsible for adding methyl groups to CpGs^2^. DNMTs are essential in mice as their deletion results in embryonic death^3^, but these embryos are nonetheless able to form all main cell types^4^.

DNA regulatory sequences, for example, promoters and enhancers, are frequently bound by transcription factors (TFs)^5,6^ and depleted of CpG methylation^7,8^. Promoter sequences are found frequently within so-called CpG islands^9^ containing a high density of CpGs that are typically unmethylated^10^ probably because of TF binding counteracting DNMTs^11–16^. DNA sequence variability in TF binding sites represents a plausible mechanism by which variation in CpG methylation arises^16–19^.

There are TFs that bind to CpG-containing motifs in DNA and some (but not all) of these TFs are influenced by CpG methylation in their binding site^20,21^. By influencing the binding sites of some TFs, CpG methylation may therefore be involved in regulating gene expression^22,23^, repeat repression^23^ and imprinting^24^. CpG methylation is nonetheless modified dynamically as a consequence of protein (for example, TF) binding to DNA^8,14,25–27^. Hence, CpG methylation is highly malleable by TFs and, for this reason, CpG methylation status is not necessarily the driving force of correlation between CpG methylation and gene expression^28–30^.

In this study, we searched for *cis*-acting influences of sequence variants on CpG methylation and explored the relevance of this DNA sequence variability to correlations observed between CpG methylation and gene expression.

## Results

### CpG methylation measured by nanopore sequencing

We performed whole-genome sequencing using nanopore technology in whole-blood samples from 7,179 individuals to at least 10× coverage (mean 20.6×; range 10–108.3×) on 8.906 PromethION flowcells, each sequenced to at least 3× coverage (mean 16.6×, range 3–39×) (Supplementary Fig. 1). 5-mCpG detection was performed on autosomes using Nanopolish^31^. CpGs were measured as units by Nanopolish if they were located within 10 base pairs (bp) of each other and, consequently, we referred to them as ‘CpG units.’ Most (83.6%) of these CpG units were ‘singletons,’ that is, represented by a single CpG site, but 16.4% of CpG units were represented by two or more CpG sites. The number of CpG units detected by Nanopolish was 22,058,476, which corresponds to 26,487,587 CpG sites in the reference genome (GRCh38).

For each CpG unit and each parental haplotype, we defined the 5-mCpG rate as the number of sequences of that parental haplotype that were methylated at the CpG unit divided by the total number of sequences covering the CpG unit on the same parental haplotype. By comparing 5-mCpG rates, as measured by nanopore sequencing, to those obtained from a subset of 132 DNA samples analyzed with oxidative bisulfite sequencing in our previous study^32^, we showed that 15.3 million CpG units were reliably detected by Nanopolish (Supplementary Figs. 2 and 3 and Supplementary Notes 1.1 and 1.2), and we confined this study to those units.

**Fig. 1:**
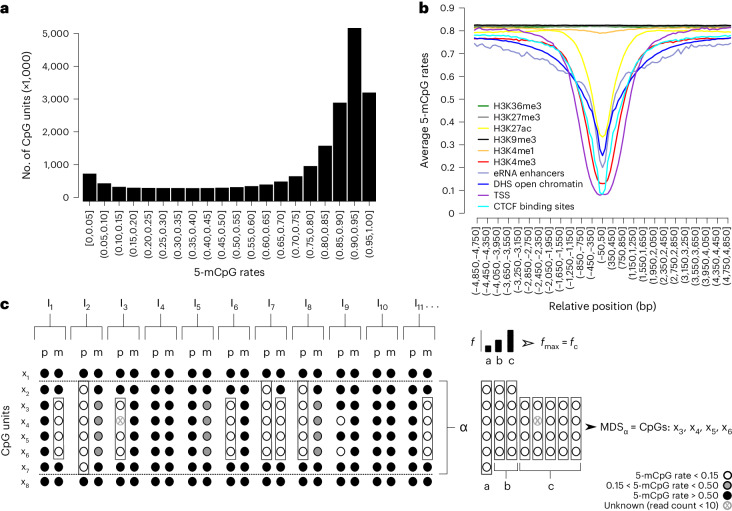
5-mCpG detected by nanopore sequencing. **a**, 5-mCpG rates computed across individuals yielded the expected bimodal distribution. **b**, 5-mCpG rates averaged in 100 bp bins relative to the midposition of chromatin makers assayed in relevant cell types, that is, histone modifications (H3K4me3, H3K27ac, H3K36me3 and H3K9me3) in primary neutrophils, CTCF protein binding sites in primary neutrophils and open chromatin regions in CD4^+^ T cells obtained from Encode and Roadmap epigenomics project. Additionally, eRNA and main TSS reference maps for RNA samples isolated from whole blood based on cap analysis of gene expression sequencing assays obtained from the Fantom5 project (SlideBase). DHS, DNase hypersensitive sites; eRNA, enhancer RNA. **c**, We applied sequence-based phasing to assign 5-mCpG status to paternal (p) or maternal (m) haplotypes in each individual (I). For each CpG unit and each parental haplotype, we computed the 5-mCpG rate and defined unmethylated haplotypes where we found three or more neighboring CpG units each with 5-mCpG rate <0.15, but located no more than 500 bp apart, in at least one haplotype (restricted to 2,648 individuals sequenced to an average coverage of >20×). A rectangle is drawn around neighboring CpG units where such unmethylated haplotypes were detected. The cluster labeled α defines locations containing overlapping unmethylated haplotypes labeled a, b and c. The most frequently occurring unmethylated haplotype (*f*_max_) is then nominated as the representative MDS of cluster α (MDS_α_) containing CpG units x_3_,x_4_,x_5_,x_6_. Source data

A bimodal distribution for 5-mCpG rates was shown (Fig. 1a), as expected given previous studies on 5-mC content in DNA and whole methylome sequencing^1^.

In this study, we classified CpG units in each individual as unmethylated (5-mCpG rate < 0.15) or low-methylated (0.15 ≤ 5-mCpG rate < 0.50). For the individual, the proportion of CpG units classified as low-methylated was, on average, 4.2% (quartiles: 3.8%, 4.5%) and the proportion classified as unmethylated was 7.2% (quartiles: 6.5%, 7.6%).

We further confirmed the well-documented lack of 5-mCpGs in functional regions mapped by Encode^33^ and others^34,35^ (Fig. 1b and Supplementary Fig. 4).

### Sequence variants associated with 5-mCpG rates

We imputed genotypes based on whole-genome sequences of 63,460 Icelanders^36^ into the 7,179 nanopore-sequenced individuals. A total of 34,435,950 high-quality sequence variants with minor allele frequency (MAF) > 10^−4^ in the sample set were located 100 kb up- or downstream of the CpG units; 23,752,296 single nucleotide polymorphisms (SNPs), 5,929,255 insertions/deletions (indels), 609,536 structural variants (SVs)^37,38^ and 4,144,863 microsatellites^39^.

We searched for *cis*-acting sequence variants (100 kb up- or downstream of CpG units) associated with 5-mCpG rates over each of the 15.3 million CpG units. Each CpG unit was tested, on average, against ~2,200 sequence variants yielding 3.4 × 10^10^ tests, and we used Bonferroni correction to set the threshold for significances at *P* < 0.05/3.4 × 10^10^, ~10^−12^.

A total of 1,625,423 CpG units associated with 1,023,970 sequence variants in a total of 1,669,151 associations; 704,474 SNPs, 205,026 indels, 106,743 microsatellites and 6,727 SVs; 263,403 (25.7%) of these sequence variants associated with more than one CpG unit. Out of the 1,625,423 associated CpG units, 43,728 (~2.7%) were associated with more than one sequence variant.

Most (73.4%) sequence variants identified in association with CpG methylation in an external cohort^40^ were replicated in our cohort (Supplementary Note 1.3).

### 5-mCpG depleted sequences

**Extended Data Fig. 1:**
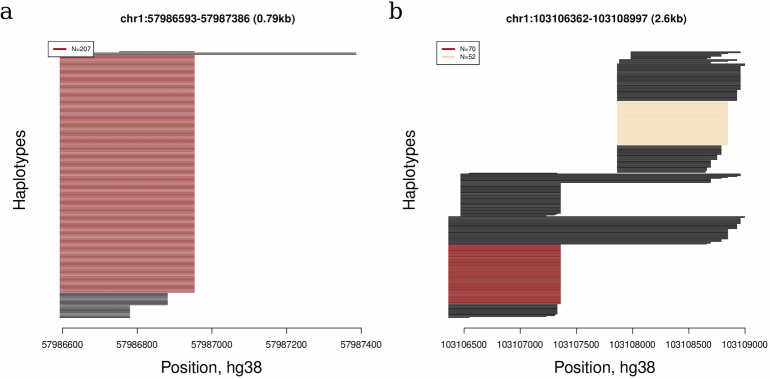
Methylation depleted sequences. Lines on the y-axis represent unmethylated haplotypes found in different individuals that overlap in position, with chromosomal position on the x axis. (**a**) For each such ‘cluster of overlapping unmethylated haplotypes’, as shown here, we look for the most frequently occurring unmethylated haplotype, shown in red. This ‘dominant’ form is then taken as the representative for the cluster of unmethylated haplotypes, which then enables comparison in 5-mCpG haplotype rates over the same coordinates (those of the representative) across all individuals in the cohort. (**b**) An example cluster where two (or more) representatives were found which we identify, and measure separately as distinct entities. chr, chromasome.

As DNA sequences depleted of 5-mCpGs are indicative of function, we searched for haplotypes where 5-mCpG rates were low across closely located CpG units in one of the two haplotypes of at least one individual (Fig. 1c). For accuracy, we confined this analysis to methylomes sequenced to an average coverage of >20× (*n* = 2,648 individuals) and CpG units where at least ten sequences were available for estimating their 5-mCpG haplotype rate. We defined unmethylated haplotypes as those with three or more CpG units each with 5-mCpG rate <0.15, but located no more than 500 bp apart. Many of the unmethylated haplotypes found in different individuals had the exact same coordinates, or were found in overlap. We therefore defined clusters of overlapping unmethylated haplotypes. For each cluster, we cataloged the genome coordinates of the most frequently occurring unmethylated haplotype (Extended Data Fig. 1a) and removed each of the unmethylated haplotypes that overlapped with these defined coordinates. If any remained, we repeated this procedure until there were no remaining unmethylated haplotypes in the cluster (Extended Data Fig. 1b). Low-methylated haplotypes were defined analogously, but with 5-mCpG rate <0.50 and only in sequences where unmethylated haplotypes were not found.

Our algorithm identified 84,924 unmethylated and 104,254 low-methylated haplotypes, hereafter referred to as methylation depleted sequences (MDSs).

Collectively, the 189,178 MDSs covered ~83 Mb of the genome and consisted of 1.2 million high-quality CpG units. MDSs were, on average, 440 bp (quartiles: 153 bp, 512 bp), and the median number of CpG units that defined each MDS was three (quartiles: 3, 4).

### Sequence variants influence the 5-mCpG rates of MDSs

We searched for *cis*-acting sequence variants (100 kb up- or downstream of MDSs) associated with 5-mCpG rates over each of the 189,178 MDSs. On average, each MDS was tested against ~2,400 sequence variants yielding 4.5 × 10^8^ tests, and we set a Bonferroni corrected significance threshold at *P* < 0.05/4.5 × 10^8^, ~10^−10^.

A total of 77,789 MDSs associated with 80,503 sequence variants in a total of 86,252 associations; 58,892 SNPs, 11,306 indels, 8,040 microsatellites and 2,265 SVs; 3,760 (4.7%) of these sequence variants associated with more than one MDS.

We refer to these 80,503 sequence variants hereafter as allele-specific methylation quantitative trait loci (ASM-QTL). Out of the 77,789 associated MDSs, 8,513 (~11%) were associated with more than one ASM-QTL. Out of 80,503 ASM-QTLs, 71,868 (89.3%) were in strong linkage disequilibrium (*r*^2^ > 0.80) to at least one of the ~1 million sequence variants found in association with 5-mCpG rates of individual CpG units.

The median distance from ASM-QTLs to the center of their associated MDS was 3.1 kb (quartiles: 0.2 kb, 16 kb). Most ASM-QTLs were common: 76,154 with MAF > 1% probably because of lack of power to detect associations with rare variants.

**Extended Data Fig. 2:**
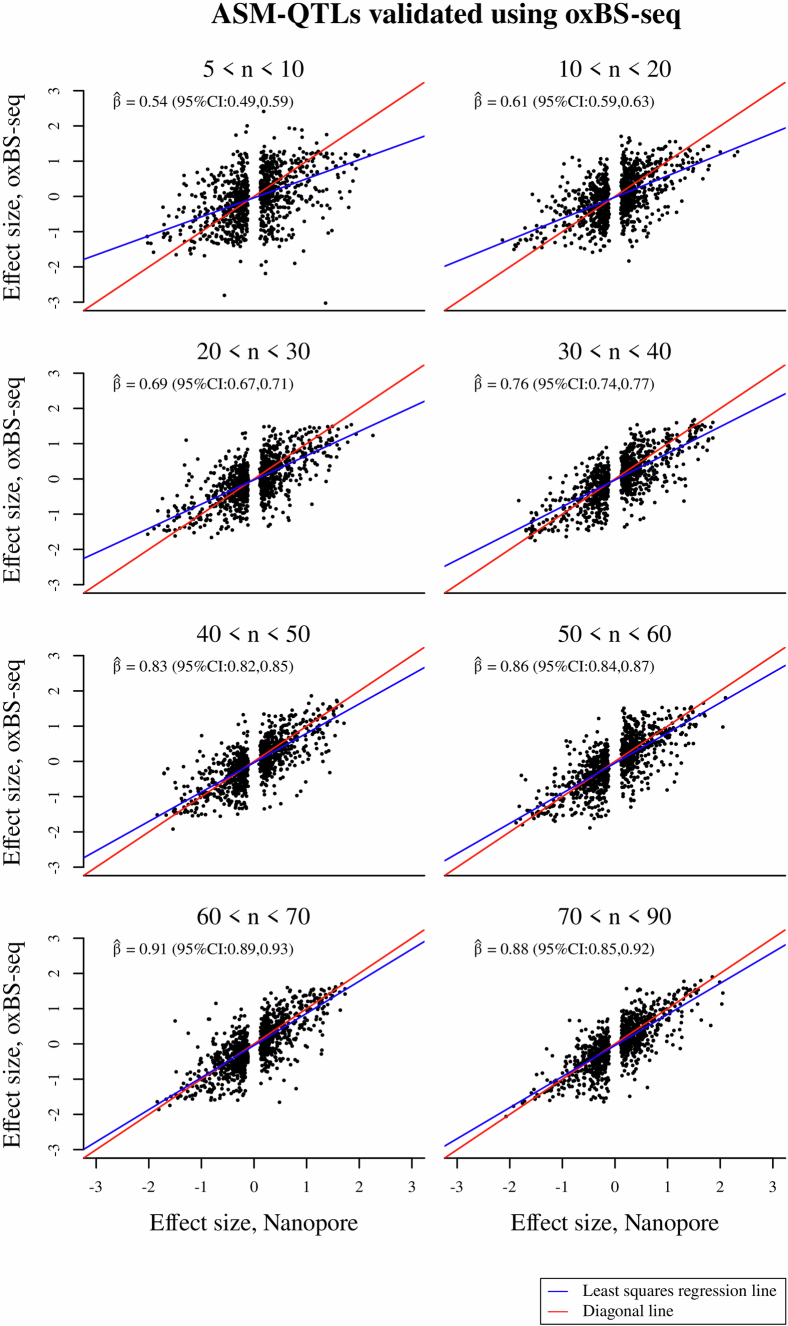
Validation of ASM-QTL influences in oxBS-seq data. ASM-QTLs validated using oxBS in independent DNA samples derived from whole blood of forty-five individuals. Note, these forty-five individuals were not included in the larger cohort of 7,179 nanopore sequenced individuals. The effect size of each ASM-QTL on the 5-mCpG haplotype rates of MDSs in oxBS sequenced individuals, y-axis, are plotted against the effect size of the same ASM-QTLs on 5-mCpG haplotype rates of the corresponding MDS in nanopore sequenced samples, x-axis. We binned the validation tests according to the number of haplotypes (n) that were available for validation testing in oxBS individuals as indicated on the top of each figure. A least squares regression line, where the effect sizes in the oxBS validation cohort are used as the outcome and those in nanopore sequenced cohort as the predictor, is shown in each figure, blue colour, along with a diagonal line of identity, red colour, as we expect the regression coefficient to be equal to one (and an intercept of zero) if the effect sizes are identical in the oxBS and nanopore sequenced individuals. The regression coefficients and their 95% confidence intervals are shown at the top left-hand corner of each figure. Note that as the number of informative haplotypes (n) in the oxBS validation cohort increases, the regression coefficient approaches that of a diagonal line indicating that the effect sizes become more similar as the number of haplotypes available for testing (n) in the oxBS cohort increases. Source data

For validation, we used whole-blood-derived DNA samples previously analyzed by oxidative bisulfite sequencing^32^, but restricted to the 45 individuals that were not included in the larger cohort used for identifying ASM-QTLs. We were able to evaluate 57,273 (out of 86,252) ASM-QTLs for association with 5-mCpG rates of the corresponding MDSs in the independent cohort of 45 individuals. Our results showed that most (89.7%; 95% confidence interval (CI): 89.5%, 90%) of the tested ASM-QTLs were consistent in effect size in the validation cohort, and the effect sizes were strongly correlated (Pearson’s *r* = 0.679; 95% CI: 0.675, 0.684) (Extended Data Fig. 2).

### Correlations between CpG methylation and mRNA expression

We performed RNA sequencing (RNA-seq) (polyA) of 896 whole-blood samples used for nanopore sequencing to analyze the effect of 5-mCpG on gene expression. The same blood samples were used to measure cellular composition and to isolate DNA and RNA for both nanopore- and RNA-seq, respectively. RNA sequences were assigned to parental haplotypes based on phase informative alleles in RNA sequence fragments. As there can be alternative transcription start sites (TSSs) for the same gene, we performed the quantification per mRNA isoform.

We searched for associations between haplotype-specific measures of 5-mCpG rates of MDSs and the haplotype-specific mRNA isoform expression of genes located 100 kb up- or downstream of each MDS. In these analyses, haplotype-specific mRNA isoform expression was represented as the proportion of mRNA sequences expressed from the paternal- and maternal haplotype. Out of the 189,178 MDSs, 83,963 were located within 100 kb from TSSs of 18,923 mRNA isoforms (9,603 genes) expressed in this collection of whole-blood samples. On average, we tested each of these MDSs against around four mRNA isoforms (quartiles: 2, 6) leading to ~380,000 tests and used Bonferroni correction to set the threshold for significance at *P* < 0.05/0.38 × 10^6^, ~1.3 × 10^−7^.

**Extended Data Fig. 3:**
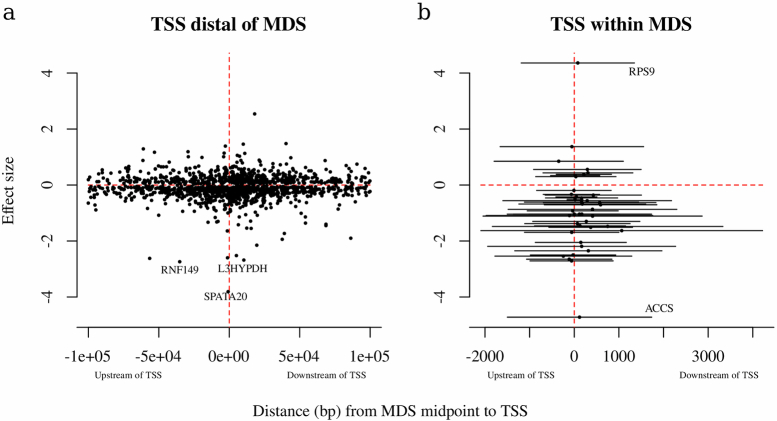
MDSs associated with gene expression. Distance, x-axis, from MDS midpoints to the TSS locations of the associated mRNA transcript isoforms *versus* the effect size on expression, y-axis. MDS midpoints are represented as black dots, and the MDSs are represented as ranges that is, horizontal lines. (**a**) Distal associations that is, where the MDS does not contain the TSS of the associated mRNA. (**b**) Promoter associations that is, where the MDS contains the TSS of the associated mRNA, where the MDS is represented by a line (left to right) indicating the position relative to the TSS of the associated mRNA. (**a**) and (**b**) TSS is represented as the origin of the x-axis (x = 0), shown as a vertical dashed line, red. Note, we modify the sign of the distance such that negative distances reflect upstream positions of the MDS midpoint relative to the TSS of the associated mRNA. Hence, the upstream positions indicate that the MDS midpoint does not intersect with the direction of mRNA transcription. In contrast, downstream positions indicate that the MDS midpoint does intersect with the direction of mRNA transcription. Source data

We found 1,103 mRNA isoforms (derived from 773 genes) in association with 957 MDSs in a total of 1,513 associations. The median distance between MDSs and the TSS of the associated mRNA isoform was 23.8 kb (quartiles: 9 kb, 47 kb). Most of the associated MDSs (921; ~96%) did not include the TSS of an associated mRNA isoform (Extended Data Fig. 3a), but 36 (~4%) contained the TSS of an associated mRNA isoform, also known as promoter methylation (Extended Data Fig. 3b).

None of these 957 MDSs overlapped with any of the previously known regions where 5-mCpG rates differ between parental chromosomes^41^, also known as imprinted regions, which was expected as our models account for parent of origin.

### ASM-QTLs correspond to TF binding sites

**Extended Data Fig. 4:**
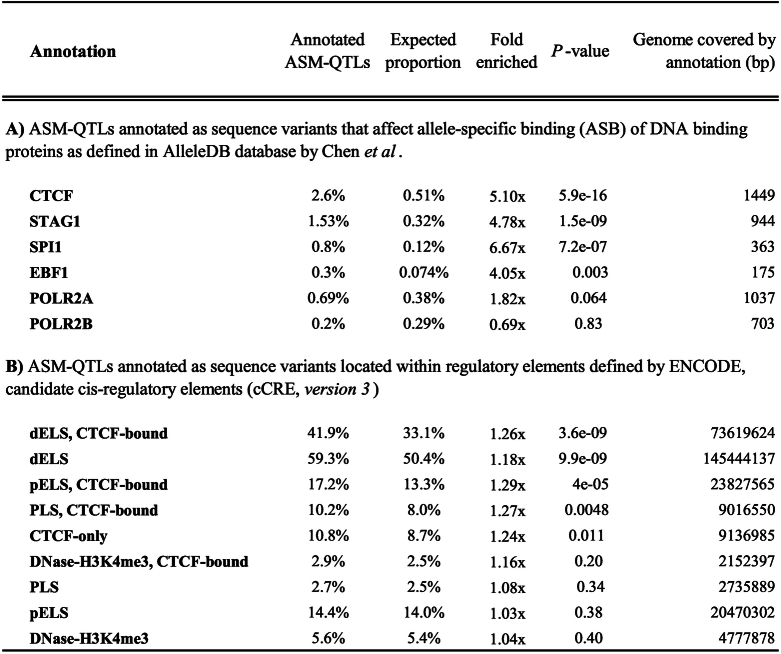
Functional attributes of ASM-QTLs. ASM-QTLs were more likely than expected by chance, to be found in high LD (*r*^2^ > 0.80) to **A**) sequence variants that influence the DNA binding affinity to transcription factors SPI1, CTCF and EBF1, and cohesin protein STAG1 and **B**) sequence variants located within regulatory sequences defined by ENCODE^33^, that is, candidate cis-regulatory elements (*version 3*). Note, sequence variants that influence ASBs were identified in Chen et al.^42^. The *P*-values shown, unadjusted for multiple comparison, were computed using our permutation-based method described under methods section „ASM-QTL in functional annotation maps‘.

The frequency of ASM-QTLs was 3.3-fold higher than expected (*P* = 8 × 10^−19^) among sequence variants found previously to influence allele-specific binding (ASB) of various proteins to DNA by Chen et al.^42^. This same database^42^ has six proteins associated with >100 sequence variants: SPI1, CTCF, STAG1, EBF1, POLR2A and POL2RB. ASM-QTLs were found more frequently (*P* < 0.05/6, ~0.008) among ASB variants for the three TFs (SP1, CTCF and EBF1) and the Cohesin Complex subunit STAG1 (Extended Data Fig. 4a).

ASM-QTLs were also more prevalent than expected among sequence variants located within regulatory elements as defined by the ENCODE project, notably within those bound by the CTCF protein (Extended Data Fig. 4b).

In accordance with previous studies^16–19^, these results support the notion that sequence variants influence methylation of CpGs through their influences on protein binding to DNA.

### ASM-QTLs dominate in correlations between MDSs and mRNA

**Fig. 2:**
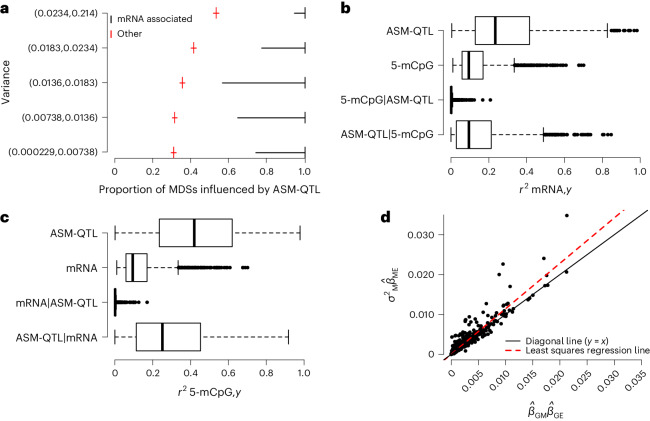
ASM-QTLs dominate in correlations found between MDSs and mRNA expression. **a**, Variance in 5-mCpG rates of MDSs, one MDS from each 100 kb segment of the genome (*n* = 25,079 MDSs), binned by quintiles (*y* axis; ~5,000 MDSs per bin) and plotted against the proportion of these MDSs that have an associated ASM-QTL (*x* axis) according to whether the MDS is associated with mRNA expression (black) or not (red). The proportion estimates are shown as tick marks (vertical lines), and their 95% CIs are shown as horizontal lines. **b**, Fraction of the variance in mRNA expression explained by each of the four variables on the *y* axis as follows: *y* = ASM-QTL represents the genotype of the ASM-QTL found in association with 5-mCpG rates of MDSs; *y* = 5-mCpG represents the 5-mCpG rates of MDSs; *y* = 5-mCpG|ASM-QTL represents the 5-mCpG rates of MDSs after correction for the ASM-QTL found in association with that same MDS; *y* = ASM-QTL|5-mCpG represents the genotype of the ASM-QTL found in association with 5-mCpG rates of MDSs after having corrected the genotype status for the 5-mCpG rates of that same MDS. **c**, Fraction of the variance in 5-mCpG rates explained by each of the four variables on the *y* axis, where mRNA represents mRNA expression but otherwise analogous to **b**. In both **b** and **c**, the center line (solid black) shown in each box represents the median; box limits represent upper and lower quartiles; whiskers represent 1.5× interquartile range. *r*^2^_mRNA,5-mCpG_ in **b** is equivalent to the reversed comparison of *r*^2^_5-mCpG,mRNA_ in **c**. **d**, Effects of ASM-QTL genotype (G) on CpG methylation (${\hat{\beta }}_{{\rm{GM}}}$) and mRNA expression (${\hat{\beta }}_{{\rm{GE}}}$), *x* axis, compared with the effects of CpG methylation on mRNA expression (${\hat{\beta }}_{{\rm{ME}}}$), *y* axis. The number of datapoints in each boxplot in **b** and **c**, and in the scatter plot in **d** corresponds to the number of associations found between methylation and gene expression (*n* = 1,513). Source data

All of the 957 MDSs that associated with mRNA expression were associated with an ASM-QTL (100%; 95% CI: 99.6%,100%). In comparison, a significantly lower proportion, that is, 40.8% (95% CI: 40.6%, 41%) of MDSs that did not associate with mRNA expression were associated with an ASM-QTL (${\chi }_{1}^{2}$ = 148.4; *P* = 4 × 10^−37^). This correspondence between MDSs associated with mRNA expression and MDSs influenced by an ASM-QTL persists irrespective of the variance in 5-mCpG rates of MDSs (Fig. 2a).

For a given MDS found in association with mRNA expression, the fraction of the variance in mRNA expression explained by the associated ASM-QTL tends to be higher (median = 0.24; quartiles: 0.13, 0.41) than that explained by the 5-mCpG rate of the MDS (median = 0.097; quartiles: 0.06, 0.17) (Fig. 2b). Further, the fraction of the variance in mRNA expression explained by the 5-mCpG rate of a given MDS is largely nullified by accounting for the effects of the ASM-QTL associated with the same MDS (median = 0.001; quartiles: 0.0002, 0.004) (Fig. 2b).

**Extended Data Fig. 5:**
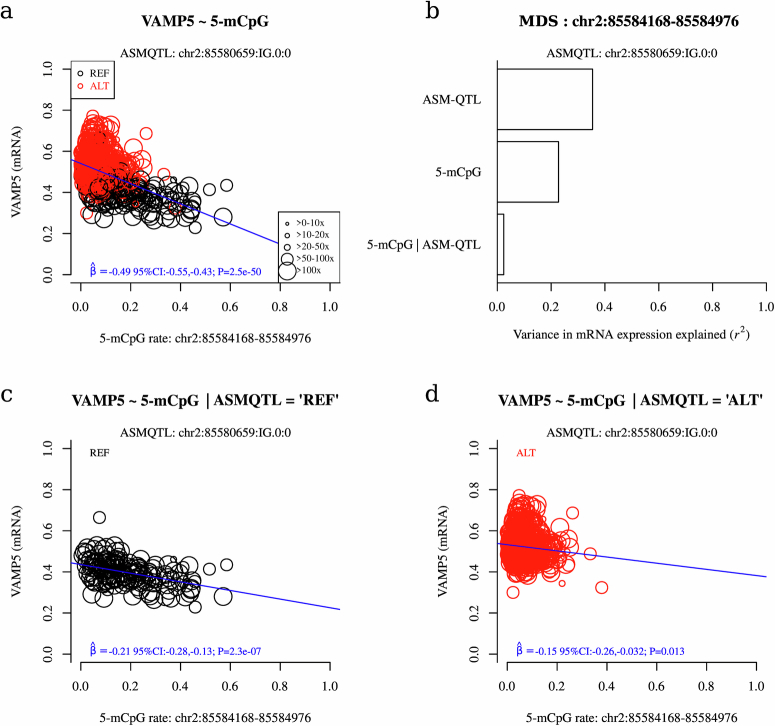
ASM-QTLs dominate in correlations between MDSs and mRNA. (**a)** Example of an association found between expression of VAMP5-201 (ENST00000306384), y-axis, and 5-mCpG haplotype rates, x-axis, of an MDS (chr2:85584168-85584976) located within the CpG island promoter of the TSS; that is, an example of CpG island promoter methylation. (**b)** The fraction of variance in VAMP5-201 mRNA isoform expression explained by (*top)* ASM-QTL found in association with the MDS *(middle)* CpG methylation of the MDS and *(bottom)* CpG methylation of the MDS after correction for the ASM-QTL. This same association between MDS at chr2:85584168-85584976 and mRNA expression of VAMP5-201 is then shown separately on (**c)** the reference- and (**d)** the alternative alleles of ASM-QTL chr2:85580659:IG.0:0, respectively. Least squares regression line is shown within each of three scatter plots, in blue colour, and the estimated regression coefficient and their 95% confidence intervals are shown at the bottom, along with the corresponding two-sided *P*-values. Source data

For example, the 5-mCpG rate of the MDS located within the CpG island promoter sequence of *VAMP5* is associated with mRNA expression of the main isoform (VAMP5-201) initiated from within that same CpG island (Extended Data Fig. 5a). The 5-mCpG rate of the MDS explains 23.7% of the variance in mRNA expression of *VAMP5-201*, whereas the ASM-QTL (1 bp deletion at chromosome 2:85580659:AT:T), associating with this same MDS, explains 35.9% of the variance in mRNA expression of VAMP5-201. After correcting the 5-mCpG rates of the MDS for the sequence variant (AT>T deletion), the fraction of the variance in mRNA expression explained by the 5-mCpG rate was only 2.5% (Extended Data Fig. 5b), suggesting that the correlation found between CpG island promoter methylation and expression of the VAMP5-201 gene is driven mostly by DNA sequence variability (Extended Data Fig. 5c,d).

The variability in mRNA expression explained by the ASM-QTL genotype status corrected for 5-mCpG rates (ASM-QTL|5-mCpG) (median = 0.09; quartiles: 0.03, 0.21) was only moderately lower than that explained by the uncorrected ASM-QTL genotype status (Fig. 2b). Similarly, the variability in 5-mCpG rates explained by the ASM-QTL genotype status corrected for mRNA expression (ASM-QTL|mRNA) (median = 0.25; quartiles: 0.11, 0.45) was only moderately lower than that explained by the uncorrected ASM-QTL genotype status (Fig. 2c).

These results indicate that sequence variants (ASM-QTLs) are responsible for creating most of the variability in CpG methylation that correlates with gene expression.

### Modeling the impact of ASM-QTL on methylation and expression

**Extended Data Fig. 6:**
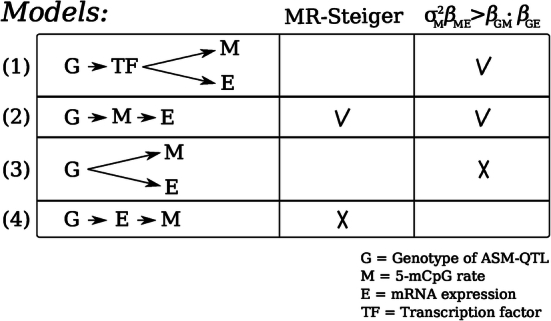
Modeling the impact of ASM-QTL on methylation and expression. Model diagrams (leftmost column) describing the four different hypotheses on the mechanism by which an ASM-QTL genotype (G) influences CpG methylation (M) and gene expression (E). The outcomes of two different tests (MR-Steiger and our method of comparing ${\hat{\beta }}_{{GM}}{\hat{\beta }}_{{GE}}$ and ${\hat{\sigma }}_{M}^{2}{\hat{\beta }}_{{ME}}$) are shown; ‘v’ shape indicates that the model is supported by the test, whereas ‘x’ indicates that the model is not supported by the test and empty cells indicates that the test is unable to consider whether or not the model is supported. For clarity, each model is labelled with a number which are then used as reference to each diagram in the main text and in Fig. 3.

We considered four different models (Extended Data Fig. 6) to infer the mechanism by which ASM-QTL variants affect CpG methylation and gene expression.

We used the Mendelian randomization–Steiger test^43^ to infer the direction of effect between the 5-mCpG rates of MDSs and mRNA expression; that is, whether 5-mCpGs are affecting mRNA levels or vice versa. Assuming equal measurement error, we found 5-mCpG rates more likely to be affecting mRNA expression for 68% of ASM-QTLs that were nominally associated with both 5-mCpG rates and mRNA expression among individuals with both measurements (*P* = 3 × 10^−41^). The measurement error of mRNA expression would have needed to be 22% greater than that of 5-mCpG rates for us to have observed this result if half of the ASM-QTLs would have supported the conclusion that 5-mCpG rates are more likely than mRNA expression to be causal. Therefore the Mendelian randomization–Steiger test provides evidence against model 4 (Extended Data Fig. 6), which states that ASM-QTLs affect 5-mCpG rates through their influences on mRNA expression, but provides support for model 2 (Extended Data Fig. 6), which states that ASM-QTLs affect mRNA expression through their influences on 5-mCpG rates.

The Mendelian randomization–Steiger test, however, does not consider horizontal pleiotropy as is present in models 1 and 3 (Extended Data Fig. 6). Model 1 states that the ASM-QTL affects the 5-mCpG rate and mRNA expression partially through a common mechanism (for example, TF binding). Model 3, however, states that the genotype (*G*) of an ASM-QTL affects the 5-mCpG rate (*M*) and mRNA expression level (*E*) through independent mechanisms. Under model 3, the product of the variance in *M* and the coefficient from regressing *E* on *M* (${\hat{\sigma }}_{\rm{M}}^{2}{\hat{\beta }}_{{\rm{ME}}}$) would be expected to be equal to the product of the coefficient from regressing *M* on *G* (${\hat{\beta }}_{{\rm{GM}}}$) and the coefficient from regressing *E* on *G* (${\hat{\beta }}_{{\rm{GE}}}$). However, we found that ${\hat{\sigma }}_{\rm{M}}^{2}{\hat{\beta }}_{{\rm{ME}}}$ is, on average, 10% greater (95% CI: 9%, 12%) than ${\hat{\beta }}_{{\rm{GM}}}{\hat{\beta }}_{{\rm{GE}}}$ (Fig. 2d), providing evidence against model 3 (Extended Data Fig. 6). The differences between ${\hat{\beta }}_{{\rm{GM}}}{\hat{\beta }}_{{\rm{GE}}}$ and ${\hat{\sigma }}_{\rm{M}}^{2}{\hat{\beta }}_{{\rm{ME}}}$ are nonetheless small, which again indicates that most of the correlation between 5-mCpG rates and mRNA expression is driven by sequence variants.

Our method of comparing ${\hat{\beta }}_{{\rm{GM}}}{\hat{\beta }}_{{\rm{GE}}}$ and ${\hat{\sigma }}_{\rm{M}}^{2}{\hat{\beta }}_{{\rm{ME}}}$ is unable to distinguish between the two remaining models 1 and 2, and the Mendelian randomization–Steiger test does not consider models 1 and 3 but provides support for model 2 over model 4 (Extended Data Fig. 6).

**Fig. 3:**
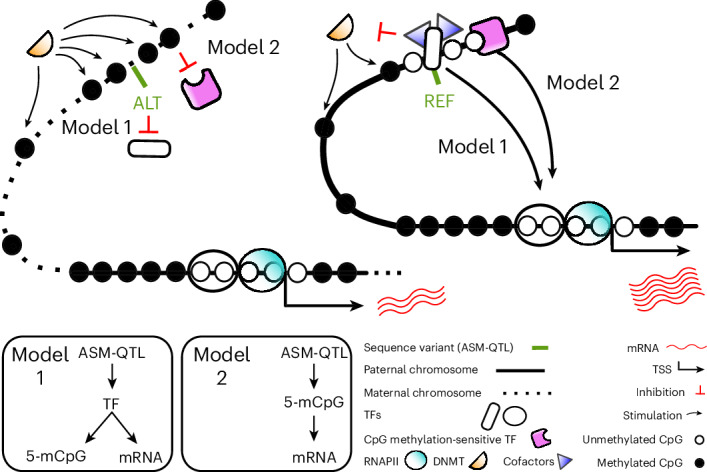
DNA sequence variability affects CpG methylation and gene expression. Illustration of the two models (models 1 and 2; bottom left) consistent with our results. In this hypothetical example, an ASM-QTL gives rise to CpG methylation differences between the maternal (left) and paternal (right) chromosomes of an individual. Under model 1, the ASM-QTL influences TF binding to DNA, which in turn influences methylation of nearby CpGs, but it is the TF (not methylation) that then results in influences on gene expression. Under model 2, the ASM-QTL influences TF binding to DNA, which again leads to influences on methylation of nearby CpGs, but here the change in methylation results in influences on gene expression, for example, by enabling binding of a CpG methylation-sensitive TF. Hence, methylation is irrelevant to gene expression in model 1 whereas it is relevant to gene expression in model 2. In both models, it is DNA sequence variability that drives the correlation between CpG methylation and gene expression. ALT, alternative allele; REF, reference allele; RNAPII, RNA polymerase II.

Our results are therefore equally consistent with both models 1 and 2, which we illustrate using a hypothetical example in Fig. 3.

### ASM-QTL enrichment among trait-associated sequence variants

We have previously performed genome-wide association (GWA) studies (GWAS) on a large number of human diseases and other traits in the Icelandic population^36,44,45^. GWAS signals identified in these studies allowed us to quantify the contribution of ASM-QTLs to human phenotypic diversity relative to other types of sequence annotations in the same model. We searched for and identified 5,071 GWAS signals (*P* < 1 × 10^−9^) in a selected list of 261 diverse traits, 60 diseases and 201 other traits.

**Extended Data Fig. 7:**
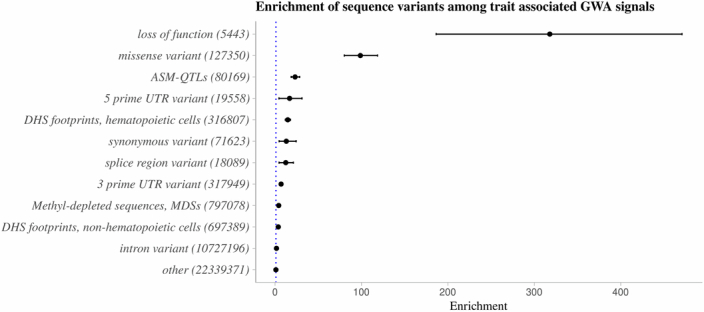
Varied contribution of sequence variants to human trait variability. Enrichment, x-axis, of ASM-QTLs and other sequence variant annotations, y-axis, among GWA signals found in various human diseases and other traits. The solid points (black) represent the measure of center, that is, the enrichment point estimates and the horizontal lines (black) represent their 95%CIs. The number of sequence variants in each annotation is shown within parentheses on the y-axis. Shown are the enrichment point estimates, points, and their 95% confidence intervals, horizontal lines, for each sequence variant annotation, and the point of neutral enrichment on the x-axis as vertical line, blue. UTR=Untranslated Region, DHS=DNase hypersensitivity site, MDSs=Methylation depleted sequences. Source data

ASM-QTLs were enriched 23.2-fold among GWAS signals (95% CI: 18.5, 28.4; *P* < 0.0001; Extended Data Fig. 7 and Supplementary Note 1.4). For comparison, in this same model, we also specified an annotation of sequence variants that resided within the MDS coordinates, which yielded 4.2-fold enrichment (95% CI: 3.2, 5.3; *P* < 0.0001) showing that location alone did not capture all the effects of ASM-QTLs. We noted that ASM-QTLs were more enriched among GWAS signals than any other noncoding annotation such as sequence variants located in DNase hypersensitivity footprints^6^ (Extended Data Fig. 7). Only protein coding variants were more enriched than ASM-QTLs, that is, missense (98.8-fold; 95% CI: 81.1, 118.5; *P* < 0.0001) and loss of function variants (292-fold; 95% CI: 178, 426; *P* < 0.0001).

**Fig. 4:**
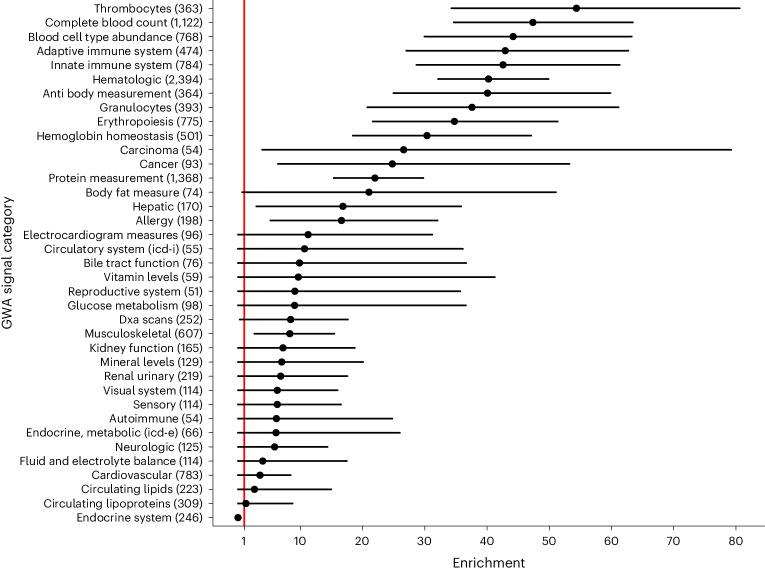
ASM-QTLs are enriched among GWA signals. Enrichment of ASM-QTLs among GWA signals varies in magnitude by trait category. The number of GWA signals for each trait category is shown in parentheses. Solid points, measure of center (enrichment point estimates); horizontal lines, 95% CIs; vertical red line, point of neutral enrichment (*x* = 1). Source data

The enrichment of ASM-QTLs varied according to the classification of GWAS signals into trait groups (Fig. 4). Notably, our ASM-QTLs were 40.2-fold (95% CI: 32.2, 49.9; *P* < 0.0001) enriched among 2,394 GWAS signals found in association with 56 hematological traits (out of 261 traits), which was higher than the overall 23.2-fold enrichment (*P* < 0.05) (Fig. 4). In contrast, ASM-QTLs were 6.6-fold (95% CI: 3.9, 9.7; *P* < 0.0001) enriched among the remaining 2,677 GWAS signals found in 205 nonhematological traits. This demonstrates that the ASM-QTLs identified here have more effects on hematological traits than on other traits, probably because of tissue-specificity in CpG methylation as our measurements were done using DNA from whole-blood samples.

ASM-QTLs found in linkage disequilibrium with sequence variants that associated with gene expression in our in-house RNA-seq data^46^, also known as *cis*-expression QTLs, were enriched 69.8-fold (95% CI: 50.6, 90.8; *P* < 0.0001) among GWAS signals. Furthermore, ASM-QTLs that were not found in linkage disequilibrium with *cis*-expression QTLs were enriched 16.8-fold (95% CI: 12.5, 21.6; *P* < 0.0001). Hence, ASM-QTLs were enriched among sequence variants relevant to trait diversity irrespective of whether they were also identified as *cis*-expression QTLs. We postulate that the ASM-QTLs that did not appear to influence gene expression may still be functionally important because their impact on expression may be context-specific, for example, only relevant after specific stimuli or environmental cues.

The ~1 million sequence variants found in association with 5-mCpG rates of individual CpG units were also enriched among GWAS signals (7.3-fold, 95% CI: 6.3, 8.3; *P* < 0.0001), which was significantly lower than the corresponding 23.2-fold enrichment for ASM-QTLs associated with 5-mCpG rates of MDSs (*P* < 0.05). These sequence variants were enriched 10.4-fold (95% CI: 8.7, 12.5) and 4.7-fold (95% CI: 3.9, 5.7) among GWA signals associated with hematological and nonhematological traits, respectively. Thus, sequence variants associated with 5-mCpG rates of MDSs have greater functional relevance than those associated with individual CpG units, showing that measurements of 5-mCpG rates within MDSs are highly informative of functional activity in DNA and relevant to human trait diversity.

### ASM-QTLs correspond to disease-associated sequence variants

Previous studies have found that sequence variants associated with human traits overlap those associated with CpG methylation^47,48^. Our results show that 964 and 4,391 ASM-QTLs were in strong linkage disequilibrium (*r*^2^ > 0.80) with sequence variants associated with 152 diseases and 431 other traits, respectively, based on published GWAS^49^.

For example, on chromosome 2q33.3, rs34329895, associating with type II diabetes^50^, is in strong linkage disequilibrium (*r*^2^ = 0.97) with the ASM-QTL rs35735821 found in association with promoter methylation of the *PLEKHM3* gene. In this example, the disease-associated variant is not found in linkage disequilibrium to a protein coding variant or any *cis*-expression QTLs. Here, ASM-QTLs offer a valuable complement to other sequence variant annotations in identifying candidate gene targets.

Another example on chromosome 10p15.1, rs12722502, associating with asthma^51^, was found in nearly perfect linkage disequilibrium (*r*^2^ = 0.997) with ASM-QTL rs12722547, which associated with 5-mCpG rates of an MDS residing within a cCRE enhancer element, intronic of *IL2RA*. Neither the ASM-QTL nor the disease-associated sequence variant were in linkage disequilibrium to any protein coding variants. Further, in our RNA-seq datasets, rs12722502 was not found in strong linkage disequilibrium (*r*^2^ > 0.8) with any conditionally independent *cis*-expression QTLs in RNA isolated from whole-blood samples from ~17,800 individuals^46^. Whereas neither RNA nor protein coding annotations provided clues to a functional consequence, the ASM-QTL points to a candidate regulatory element, which can then be investigated further in experimental models.

## Discussion

In this study, we assigned CpG methylation, gene expression and alleles of sequence variants to parental haplotypes, allowing us to investigate correlations between the three sets of measurements on a haplotype level. We used these data to identify MDSs and found that, in instances where their CpG methylation correlated with gene expression, a sequence variant was invariably found in association with the CpG methylation of the same MDSs that explained most of the correlation. Hence, in instances where CpG methylation is found in association with both a sequence variant and gene expression, it is important to be cautious about assuming that the sequence variant influences the gene expression through CpG methylation. Indeed, our results are consistent with a model in which the correlations found between CpG methylation and gene expression are mostly byproducts of variability in TF protein binding to DNA created by sequence variants. In this model, TFs, but not CpG methylation, are responsible for influencing gene expression. Nonetheless, our results are equally consistent with a model in which the sequence variant exerts its influences on mRNA expression by affecting CpG methylation. In both models, however, the sequence variant is the primary driver of the correlation between CpG methylation and gene expression.

We show that sequence variants found in association with variation in CpG methylation rates of MDSs have substantial effects on human phenotypic diversity. Previous studies have described haplotype-specific influences of sequence variants on CpG methylation^16,17^. However, due to small sample sizes they lacked power to carry out association analyses designed to detect consistent allele-specific influences across individuals and, as such, were not well suited to evaluate the effect of these sequence variants on human phenotype diversity.

Limitations of our study include variability in cell-type composition, which we accounted for by using a statistical technique—singular value decomposition—while also using information on direct measurements of cell-type composition available in a subset of our cohort. We note, however, that both methods are limited in resolution of specific subpopulations of blood cell types and therefore account incompletely for cell-type composition. We would also like to note that additional insights may be gained into the relevance of CpG methylation to human diseases, and other traits, by using genetic colocalization methods^52^ instead of the linkage disequilibrium approach used here. Also, the mapping of *cis*-expression QTLs is an ongoing effort involving contributions from numerous entities around the world, which therefore complicates our ability to declare whether or not a sequence variant has been identified as a *cis*-expression QTL, as the field is evolving continuously. Finally, as we accounted for parent of origin in our models, our results do not apply to gene imprinting wherein 5-mCpGs may have important regulatory roles^53^.

Nanopore sequencing provides accurate detection of CpG methylation in DNA samples and has the benefit of achieving long sequences that facilitate phasing of the sequences to parental chromosomes to yield haplotype resolved methylomes (Supplementary Note 1.5). Measurement of CpG methylation in DNA samples allows for evaluation of protein binding and the effect of sequence variants on protein binding. CpG methylation has many properties that make it more suitable than other chromatin-based assays for functional annotation of the noncoding genome. This includes its chemical stability and its measurement accuracy, which ensures comparability between samples from different individuals. We expect nanopore sequencing of genomes from various cell types and tissues will be instrumental to investigating noncoding sequence variants of functional relevance.

## Methods

### Ethical statement

This study was approved by the National Bioethics Committee in Iceland (approval no. VSN 14-015) and conducted in agreement with instructions issued by the Data Protection Authority in Iceland (PV_2017060950ÞS/--). All individuals gave informed consent, and all personal identifiers were encrypted by an external agent before being imported into the deCODE database.

### Statistics and reproducibility

In this study, we nanopore sequenced DNA isolated from whole-blood samples from 7,179 Icelanders (3,434 male, 3,745 female) participating in various studies at deCODE genetics^36,44,45^. The earliest year of birth was 1876 and 1890 for male and female participants, respectively, and the latest was 2015 for both sexes. The median year of birth was 1960 for male and 1958 for female participants.

No statistical method was used to predetermine sample size. The sample size was determined based on the number of nanopore-sequenced DNA samples. We excluded nanopore-sequenced DNA samples derived from tissues other than whole blood. We further excluded individuals where we obtained less than ten times nanopore sequencing coverage after restricting to flowcells sequenced to at least three times average coverage, as described in ‘Results’. No animals were used in the study.

### DNA isolation and sequencing

DNA from whole blood was extracted using the Chemagic method (PerkinElmer)—an automated procedure that involves the use of M-PVA magnetic beads. Quantitation of genomic DNA was performed on the Big Lunatic instrument using software and plates from the manufacturer, and the absorbance ratio for quality. DNA integrity was assessed using the Fragment Analyzer capillary system from AATI, following the manufacturerʼs guidelines.

DNA sequencing libraries were generated using the SQK-LSK109 ligation kit from ONT. Sample input varied from 1 to 5 μg DNA, depending on the exact version of the preparation kit and the flowcell type used for the PromethION sequencing. Nanopore sequencing was performed using PromethION machines, using R.9.4 flowcells, following ONT standard operating procedures. Data acquisition varied from 48 to 60 h per flowcell.

### CpG methylation detection by nanopore sequencing

Squiggle data from the sequencers were basecalled using Guppy and mapped to the human reference genome GRCh38 with Minimap2 (ref. ^54^). We then used Nanopolish^31^ to detect 5-mCpG from nanopore-sequenced DNA. Nanopolish detects methylated cytosines in a CpG context using a Hidden Markov model to assign a log-likelihood ratio for the presence of a cytosine methylation at each CpG site. We interpret values above +1.921 as indicating support for cytosine methylation and less than −1.921 as support for unmethylated CpG. Nanopolish groups CpG sites within 10-bp distance and assigns a methylation status to each group such that all CpG sites within a group have the same methylation status. For this reason, we refer to CpG sites measured by Nanopolish as CpG units. 5-mCpG status was assigned as unreliable if the prediction was ambiguous (−1.921 ≤ log-likelihood ratio ≤ 1.921).

We assessed the impact of various attributes of the CpG units on the quality of 5-mCpG detection (Supplementary Notes 1.1 and 1.2). First, strand bias and the fraction of reliable reads (FRR) was calculated for each CpG unit by averaging over the whole dataset. We then defined strand bias as the difference in methylation levels between forward and reverse strand, and FRR as the fraction of reliable reads out of all reads. CpG units were removed if the strand bias was ≥0.20 or if the FRR was ≤0.5. Second, we removed CpG units within 5 bp of a known SNP locus (MAF > 0.001) as the presence of a sequence variant in a pore at the same time as an unmethylated CpG may produce an electric signal similar to the signal of 5-mCpG, and vice versa. Third, we removed CpG units of coverage higher than 1.5 times the average as this is evidence of repetitive regions, CpG units of coverage lower than 0.5 times the average as this indicates SVs or regions where the sequencing or mapping might be problematic. Fourth, we removed CpG units located within so-called dark regions^55^ of the genome as these regions contain sequence repeats that cause mapping to be unreliable. Fifth, we removed CpG units where the fraction of phased sequences was less than 0.3 as this is indicative sequences that are difficult to phase. In total, 15,317,794 CpG units satisfied our criteria for high-quality detection of 5-mCpG in nanopore-sequenced DNA samples.

We restricted our cohort to DNA samples from whole blood that were nanopore-sequenced to at least ten times coverage analyzed on flowcells with at least three times coverage (Supplementary Notes 1.1 and Supplementary Figs. 1 and 2). The mean N50 (measure of average sequence length) of our nanopore sequence data is 18,861 bp (median = 18,376; minimum = 4,491, maximum = 50,719) (Supplementary Fig. 1).

We define coverage as the total number of sequenced base pairs divided by 3 × 10^9^, approximately the size of the reference genome.

### Assignment of 5-mCpG status to parental haplotypes

DNA sequence variants were called using Graphtyper^56^ based on 63,460 whole-genome sequenced individuals representing a subset of 173,025 SNP chip-typed individuals from the Icelandic population^36–39^. Whole-genome sequencing was carried out using Illumina sequencing to mean depth of 39.8× (range 20–397.8×). The sequences were then phased to impute haplotypes into the chip-typed individuals.

Whole-genome sequences of individuals in our study had been long-range phased and assigned parent of origin^57^, enabling us to assign sequences analyzed by Nanopolish to maternal or paternal chromosomes. Parental haplotypes were assigned by examining the phasing status of a set of 8,960,728 high-quality sequence variants^58^, using heterozygous carriers of in-read sequence variants, allowing us to assign CpG methylation calls (methylated or unmethylated) to maternal and paternal chromosomes. Sequences overlapping at least three heterozygous variants and where at least 70% of the variants where consistent with the phasing of one parent were considered to be phased and used for subsequent analysis.

For each CpG unit and each parental haplotype, we defined the 5-mCpG haplotype rate as the number of sequences of that parental haplotype that are methylated at the CpG unit divided by the number of parental sequences covering the CpG unit.

### Methylation depleted sequences

MDSs are defined as sequences depleted of 5-mCpGs across at least three CpG units located within 500 bp of each other, on the same haplotype in at least one individual.

We applied measurements of 5-mCpG rates on individual haplotypes to find instances where closely located CpG units are found depleted in methylation on the same haplotype. We confined our search for 5-mCpG depleted haplotypes to nanopore methylomes sequenced to an average coverage of >20× (*n* = 2,648 individuals) and we restricted to CpG units whereat least ten sequences were available for estimating the 5-mCpG rate. For each individual and each of its parental haplotypes, we defined unmethylated haplotypes as the occurrence of three or more CpG units on the same haplotype, each of which displaying 5-mCpG rates <0.15, but located no more than 500 bp apart from each other. Unmethylated haplotypes found in different individuals often shared the exact same coordinates, that is, they were defined by the exact same CpG units, or varied slightly from one individual to another. We therefore defined clusters of overlapping unmethylated haplotypes based on CpG position; unmethylated haplotypes sharing one or more CpG units were clustered together. In each such cluster, we restricted to unmethylated haplotypes where the CpG units located immediately up- and downstream were measured, that is, based on more than ten sequences. We then cataloged the genome coordinates of the most frequently occurring unmethylated haplotype. We then removed unmethylated haplotypes found in overlap to these coordinates, to then determine whether there were any remaining unmethylated haplotypes in that same cluster. If so, we repeated the process of cataloging the most frequently occurring unmethylated haplotype until the cluster was emptied. In instances where there was more than one unmethylated haplotype with the highest frequency, we cataloged the coordinates of the longest (bp) haplotype amongst them.

Low-methylated (but not unmethylated) sequences have been shown to be a characteristic feature of distal regulatory elements^8^. We therefore defined low-methylated haplotypes as the occurrence of more than three CpG units on the same haplotype, each with 5-mCpG rate <0.5, but only in sequences where unmethylated haplotypes were not found. We refer to the collection of unmethylated- and low-methylated haplotypes as MDSs.

We then measured the methylation level of each MDS by counting the number of methylated and unmethylated CpG units positioned within each MDS on each parental haplotype in each individual to then compute the 5-mCpG rate: the number of methylated CpG units divided by the number of methylated and unmethylated CpG units. Hence, even though we restrict to a subset of individuals to search for MDSs, we still measure the 5-mCpG rate of MDSs in all 7,179 individuals in our cohort.

### Allele-specific methylation quantitative trait loci

ASM-QTLs are defined as alleles of sequence variants that lead to local changes in the methylation status of CpG sites on the same inherited sequence.

To identify ASM-QTLs, we followed a two-phased procedure. In the first phase, we used least squares regression to identify the most likely causal variant. In the second phase, we removed variants that did not associate after we conditioned on cellular composition among the 1,934 of 7,179 (27%) individuals where we had this information.

In Phase 1, we defined a total of 34,435,950 high-quality sequence variants^36–39,56^ with MAF > 10^−4^ by filtering on sequence variants with imputation information above 0.9, alternative allele score (SNP/indels only) above 0.5, average allele balance of heterozygous and homozygous individuals above 27.5% and 96.5%, respectively, root-mean-square mapping quality of the overlapping reads above 20, between 10% and 90% of the overlapping reads mapped on the forward strand, at least five reads supporting the alternative allele in each individual and at least 30% of the reads supporting the alternative allele in any individual.

These criteria yielded 23,752,296 SNPs^56^, 5,929,255 indels^56^, 609,536 SVs^37,38^ and 4,144,863 microsatellites^39^.

We used multivariate least squares regression (fastLm function in RcppEigen^59^ package v.0.3.3.9.4; R v.3.6) to search for sequence variants associated with 5-mCpG rates of each of the ~189,000 MDSs measured on each of the two haplotypes in the 7,179 individuals in our cohort. We set a Bonferroni corrected significance threshold in accordance with the number of hypothesis tests performed (*P* < 0.05/4.5 × 10^8^, ~10^−10^). In each association signal, the most significant variant was selected as the ASM-QTL (representing the likely causal variant); also referred to as the ‘primary’ association.

As the distribution of 5-mCpG rates cannot be assumed to be normal for each and every MDS, we performed the inverse normal transformation.

For each MDS, we restricted the search to sequence variants located 100 kb up- or downstream of the MDS. We included measured haplotypes regardless of the number of sequences used to estimate the 5-mCpG rate of the MDS to perform the test for association with alleles of sequence variants, but we excluded MDSs where fewer than 100 haplotypes were measured.

Covariates were as follows: age, sex (male, female), parental haplotype (paternal, maternal), and the first five principal components computed on all autosomes apart from the autosome containing the MDS being tested for association, see further definition in the Covariates section below.

In searching for ‘secondary’ associations (that is, to see whether there are more than one ASM-QTLs for each MDS), we confined our search to the major allele of the primary association variant for each MDS, and then performed the same association analysis accounting for the same covariates and, as before, used the same *P* value threshold at <10^−10^ for detecting secondary associations and, also as before, we then selected the most significant sequence variant as the index for the secondary ASM-QTL.

In Phase 2, information on cell-type composition was available for 1,934 (out of 7,179) individuals measured in the same blood draws as were used to isolate the DNA for nanopore sequencing. We restricted the set of ASM-QTL to those that remained significant at an false discovery rate of 0.5% after accounting for cell-type composition in the subset of 1,934 individuals. We did this by adding cell count information to the covariates in Phase 1. We included counts of neutrophils, basophils, eosinophils, immature granulocytes, monocytes, white blood cells and red blood cells in addition to the fraction of nucleated red blood cells. We did not include lymphocyte counts because of their strong correlation with neutrophil counts (Pearson’s *r* = −0.94) to avoid colinearity in the regression.

We then followed the same two-phased procedure to identify sequence variants associated with individual CpG units.

### Covariates

We sampled two random subsets of the CpG methylation data: one used for training and the other for testing. Each subset consisted of approximately 1% of the 15.3 million high-quality autosomal CpG units. By using an ‘add one’-based method we tested the CpG units for association with the following covariates: quality control measures (N50, number of ultra long sequences, percentage alignment and percentage error) and sequencing measures (sequencing device, concentration, ratio, source type and storage time).

This association analysis was carried out as follows: first, we ran association of all the covariates to each site in the training subset and ranked the covariates based on their median *P* value. Second, using the ordering from the first step, we compared the goodness of fit for the model containing *n* covariates with the model containing *n* + 1 covariates for each site in the test set, where 0 ≤ *n* ≤ *m*, with *m* denoting the total number of covariates. We continued until adding more covariates no longer yielded a significantly better model for most sites at a nominal *P* value threshold of <0.05.

Using these criteria, we chose not to adjust for any of these covariates as the first ordered covariate was significant for only <10% of the sites.

We sampled a random subset of the methylation data and computed principal components (PCs) for each autosome separately by using CpG units from all other autosomes (Supplementary Fig. 5). We chose to adjust for the first five PCs as they collectively explain approximately 2.84% of the variance in 5-mCpG rates (Supplementary Fig. 6); each of the other PCs explain less than 0.12%, and were therefore omitted.

Currently, methods used for predicting cell-type counts for array-based measurements of methylation have not been validated for nanopore sequencing data. We therefore accounted for cell-type composition by using the first five PCs as they capture a large fraction of the variability in neutrophils and lymphocytes (Supplementary Fig. 5), while also using direct measures of cell-type composition in individuals available for a subset of our cohort, see sections ‘Allele-specific methylation quantitative trait loci’ and ‘RNA-seq and phasing to parental chromosomes.’

### ASM-QTLs validated in an independent cohort

Validation of ASM-QTLs found in association with 5-mCpG rates of MDSs was performed using oxidative bisulfite sequencing performed in our previous study^32^ using DNA samples isolated from whole-blood samples from 45 individuals. Note, these 45 individuals used for validation were not included in our cohort of 7,179 nanopore-sequenced individuals and were therefore independent of the study cohort. We performed the same multivariate least squares regression as was performed for nanopore-sequenced samples with age, sex and parental haplotype as covariates in both oxBS and nanopore sequencing data. As a measure of consistency between the ASM-QTL effect sizes in the 45 and 7,179 individuals sequenced by oxBS and nanopore sequencing, respectively, we computed:

$$t=\frac{{\hat{\beta }}_{{\rm{ox}}}-{\hat{\beta }}_{{\rm{nano}}}}{\sqrt{{s}_{{\rm{ox}}}^{2}+{s}_{{\rm{nano}}}^{2}}}$$

Here, $\hat{\beta }$ represents the effect size estimates for ASM-QTLs and *s*^2^ is the variance of those effect size estimates in oxBS (ox) and nanopore (nano) sequenced individuals. The proportion of consistent effect sizes was then computed as the number of nominally nonsignificant differences between the ASM-QTL effect sizes in oxBS and nanopore-sequenced individuals (*P* > 0.05) divided by the total number of ASM-QTLs that we were able to test for validation in the 45 oxBS sequenced individuals. The *P* value was based on the *t*-statistic with *n* − 1 degrees of freedom where *n* is the number of informative haplotypes in the regression analysis in the oxBS validation cohort.

### ASM-QTL in functional annotation maps

We tested whether ASM-QTLs were more likely than expected by chance to be identical, or in high linkage disequilibrium, to sequence variants identified as functionally relevant in other studies^33,42^.

ASM-QTLs, or sequence variants in high linkage disequilibrium (*r*^2^ > 0.80) to ASM-QTLs, are often found in close proximity to one another and may therefore be found in the same annotated region. To eliminate such dependencies between observations due to proximity we employed the following procedure: first, we selected the single most significant ASM-QTL (based on the lowest *P* value). Second, we selected the most significant of the remaining ASM-QTLs that was at least 1 Mb away from the already selected ASM-QTL. Third, we repeated step two until no more ASM-QTLs could be found at least 1 Mb away from those ASM-QTLs already selected. In this way, we obtained a subset of ASM-QTLs (*n* = 1,929), hereafter referred to as the ‘observed ASM-QTLs,’ for use in analysis of enrichment among functional annotation maps of the genome.

The proximity to MDSs and the number of sequence variants found in high linkage disequilibrium (*r*^2^ > 0.80) to the 1,929 observed ASM-QTLs are expected to influence the probability of finding an overlap with functional annotations of the genome. We therefore sampled the same number of sequence variants, that is 1,929, from regions located <10 kb from the midpoint of any of the 189,000 MDSs, while also ensuring that the 1,929 sampled variants are matched to the each of the 1,929 observed ASM-QTLs with respect to the number of sequence variants found in high linkage disequilibrium. Additionally, we require that the 1,929 sampled variants are spaced by >1 Mb as this was also the requirement for the 1,929 observed ASM-QTLs. We refer to a sampled variant and variants found in high linkage disequilibrium to the sampled variant as a ‘sampled signal.’ We then count the number of sampled signals that overlap with sequence variants found within a functional annotation (this count is denoted as *z*). This procedure is then repeated *N* = 50,000 times. In summary, we are simulating the ASM-QTLs in terms of (1) the proximity of ASM-QTLs to MDSs and (2) the number of sequence variants in high linkage disequilibrium to the ASM-QTLs.

Let *z*_*i*_ represent the number of sampled signals that were annotated in each *i*-th set of *N* samples. The probability that a sampled signal overlaps a functional annotation is then:

$$P=\frac{\mathop{\sum }\nolimits_{{i}}^{N}{z}_{{i}}}{{nN}}$$

Here, *n* = 1,929 is the number of sampled variants and *N* = 50,000 is the number of sampled sets.

To determine the probability of observing *x* or more ASM-QTLs in a given annotation, where *x* is the observed number of ASM-QTLs that overlap with that annotation, we compute $P\left(X\ge x\right)=j/N$, where *j* is the number of times we found *x* or more of the 1,929 sampled signals in overlap with the annotation in each of the aforementioned *N* = 50,000 sampled sets.

In instances where *j* = 0, we compute the *P* value using a binomial approximation.

We define *X* as a random variable that follows a binomial probability distribution Bin(*n*, *p*) representing the number of ASM-QTLs found in a functional annotation. As we have ensured a minimum of 1 Mb distance between the observed ASM-QTLs we assume that the observations are largely independent. We then use the probability density function of the binomial distribution for Bin(*n*, *p*) to compute *P*(*X* ≥ *x*) as the sum of probabilities of finding *x* or more ASM-QTLs in the functional annotation using the dbinom function in R.

The fold enrichment is computed as: $\frac{p{\prime} }{p}$ where $p{\prime} =\frac{x}{n}$

### ASM-QTL enrichment among trait-associated sequence variants

In this study, a GWA signal is defined as the lead (strongest) association variant for a human trait and sequence variants found in high linkage disequilibrium (*r*^2^ > 0.80) to this same lead variant. We used GWA signals (*P* value < 10^−9^) identified in a diverse set of 261 human phenotypes in the Icelandic population^36,44,45^, including 60 diseases and 201 other traits.

We restricted our search to the ~34.4 million sequence variants that we used to search for ASM-QTLs (section ‘Allele-specific methylation quantitative trait loci’). We selected the strongest associating variant (the highest *χ*^2^) within each 1-Mb interval to make a list of candidate association variants. For each trait, we then retained only sequence variants that are associated independently with the trait that is only the strongest association variants are retained from each chromosome if they still associate with the same trait at *P* < 10^−9^ after correcting for other candidate association variants located on the same chromosome.

We defined the enrichment among GWA signals as the fold change in the proportion of GWA signals among sequence variants of a given annotation category relative to the genome-wide proportion of variants in that category.

We used our previously described model for estimating enrichment of sequence variant annotation among GWA signals^44^, but with modifications that were critical for enabling analysis of annotations that are skewed towards common variants, as are our ASM-QTLs. In ref. ^44^, the enrichment of an annotation *c*, *E*_c_, was estimated as $\frac{{P}_{\rm{c}}}{{q}_{\rm{c}}}$, where *P*_c_ is the probability of a causal variant being from annotation c and *q*_c_ is the probability of a noncausal variant being from annotation *c*. The derived approximate likelihood over all association signals *i*, each with marker set *M*_*i*_ was as follows:

$${\mathcal L} ({{P}})=\prod _{{i}}\sum _{{m}\in {{M}}_{i}}{e}^{{\chi }_{\rm{m}}^{2}}{P}_{{\rm{c}}_{\rm{m}}}\prod _{{m}{\prime} \ne {m}}{\hat{q}}_{{\rm{c}}_{m{\prime} }}$$

Here, ${{{\chi }}}_{\rm{m}}^{2}$ is the test statistic from the GWA for an individual sequence variant *m* and *c*_m_ is the annotation of variant *m* and ${\hat{q}}_{\rm{c}}$ is an estimate of *q*_c_ as the proportion of variants coming from annotation c among tested variants. The parameters *P*_c_ were inferred by maximizing the above likelihood and the enrichment estimates are then ${\hat{E}}_{\rm{c}}=\frac{{\hat{P}}_{\rm{c}}}{{\hat{q}}_{\rm{c}}}$.

This model works well to estimate enrichment of VEP (variant effect predictor) annotations^60,61^ but has its drawbacks in the current context. ASM-QTLs are identified on the basis of association analyses and, as a result, their MAF is skewed towards common variants. GWA signals are likewise skewed towards common variants as their detection also depends on statistical power, that is, the larger the number of individuals the more power there is for detection of associations to sequence variants in the lower end of the MAF spectrum. This skew of ASM-QTLs towards common variants will therefore have the tendency to inflate enrichment estimates among GWA signals, which makes them incomparable with enrichment estimates of other frequency independent annotations in VEP. To alleviate this problem, we modified the model such that the parameter *P*_c_, the probability of a causal variant in an association signal being from annotation *c*, is allowed to vary with variant frequency. Further, we reparametrized the model under the assumption that the true enrichment of annotation *c*, *E*_c_, is independent of variant frequency and write for frequency bin f:

$${P}_{\rm{c},f}=\frac{{E}_{\rm{c}}{q}_{\rm{c},f}}{{\sum }_{k\in C}{E}_{k}{q}_{k,\rm{f}}}$$

${q}_{\rm{c},\rm{f}}$ is the probability of a noncausal variant coming from annotation *c* in frequency bin *f* and *C* is set of annotations and *k* is a running index over annotations in *C*. The denominator of the expression above implicitly ensures that ${P}_{\rm{c,f}}$ is between 0 and 1. The modified approximate likelihood, which now is only a function of the enrichments *E*_c_, is then as follows:

$${\mathcal L} ({{E}}\;)=\prod _{{i}}\sum _{{m}\in {{M}}_{\rm{i}}}{e}^{{\chi }_{\rm{m}}^{2}}\frac{{E}_{{\rm{c}}_{\rm{m}}}{\hat{q}}_{{\rm{c}}_{\rm{m,f}}}}{{\sum }_{k\in \rm{C}}{E}_{k}{\hat{q}}_{\rm{k,f}}}\prod _{{m}{\prime} \ne {m}}{\hat{q}}_{{\rm{c}}_{\rm{m}{\prime} ,\rm{f}}}$$

${\hat{q}}_{\rm{c,f}}$ is an estimate of ${q}_{\rm{c,f}}$ as the proportion of variants coming from annotation c in frequency bin f among tested variants. We then estimated the enrichment by maximizing the above likelihood. We assumed *E*_c_ ≥ 0 for all *c* ∈ *C* and, to ensure identifiability, we set the annotation with the largest number of variants, which in this study are intronic variants, equal to 1. In other words, the largest annotation serves as a baseline and the enrichment estimates of other annotations are relative to it. We selected the frequency bins such that they included approximately the same number of association variants (~1,014 GWA signals per each of five frequency bins). All sequence variants that belonged to a GWA signal were taken to be in the same frequency bin as the strongest (lead) variant for that signal.

For further details on the derivation of the model see ref. ^44^. To validate the model modification, we carried out extensive analyses using random annotations of varying sizes and frequency distributions to show that the method is not sensitive to either frequency distribution or size of the variant annotation.

We employed the Rsolnp package in R (solnp function) to maximize ${\mathcal{L}}\left({{E}}\right)$ and obtain the estimates ${\hat{E}}_{\rm{c}}$. In practice, to handle the large numbers resulting from multiplication of the exponential of these test statistics, we input ${e}^{{{{\chi }}}_{\rm{m}}^{2}}$ in the following form: ${e}^{{{{\chi }}}_{\rm{m}}^{2}}-{e}^{{{\max }_{{\rm{m}}^{{\prime}}}}{({{\chi}}_{\rm{m}^{{\prime}}}^{2}})}$, where the maximum is taken over all *χ*^2^ statistics in GWA signal *i*.

In the models discussed in ‘Results,’ we specified 12 annotations of sequence variants based on VEP with the addition of ASM-QTLs and DNase hypersensitive site footprints^6^ as listed in Supplementary Table 1.

### RNA isolation and sequencing

Total RNA was isolated from PaxGene (QIAGEN) blood tubes using the Chemagic Total RNA Kit special (PerkinElmer). The quality and quantity of the RNA was assessed using either the Agilent BioAnalyzer (RNA 600 Nano kit) or the LabChip GX instrument (PerkinElmer) using the 96-well RNA kit.

Indexed cDNA libraries were prepared using the TruSeq RNA sample preparation v.2 kit from Illumina (96-well plate format). In short, between 0.1 and 1 µg of total RNA was used for polyA mRNA capture using oligo-dT attached magnetic beads. cDNA synthesis was done using SuperScript II (Invitrogen) and random hexamer priming. End-repair, 3′-adenylation, ligation of dual indexed adapters (IDT for Illumina), AMPure XP bead purification and PCR amplification were performed as described by Illumina. Quantity and quality of the resulting cDNA sequencing libraries was assessed using the LabChip GX, followed by standard dilutions to 3 nM. Samples were stored at −20°C in barcoded 96-well trays, with all reagent and sample handling workflows registered in an in-house laboratory information management system.

Further quality assessment was performed by doing pool sequencing (96 samples per pool) on an Illumina MiSeq instrument to optimize cluster densities and assess insert size, sample diversity and so on.

Samples were pooled, clustered on flowcells using either Illuminaʼs cBot and the TruSeq PE cluster kits (four to eight samples per pool per lane), or on NovaSeq S4 flowcells (24 samples per pool per lane) using on-board clustering, respectively. Paired-end sequencing (2 × 125 cycles) was performed with either HiSeq2500/HiseqX instruments using the TruSeq SBS kits from Illumina or NovaSeq instruments using the S4 flowcells, following the XP workflow.

### RNA phasing to parental chromosomes

We aligned RNA sequences separately to maternal and paternal genome references. The diploid haplotype reference was created for each individual and long-range phased haplotype^57^. We assigned phasing of each RNA-sequenced fragment to maternal or paternal inheritance based on higher alignment score from STAR-aligner (v.2.5.3a). We parsed fragments overlapping heterozygous variants to inspect concordant variant alleles and assigned to gene transcript based on genomic location in Ensembl v.87 (limiting to BASIC and Support II level transcripts)^62,63^. We aggregated maternal and paternal fragment counts per gene. We removed measurements for individuals with a high rate of fragment multimapping or inconsistency between fragment alignment phasing and the phase of detected allele in fragment sequence.

We tested for haplotype-specific associations between methylation and expression by fitting least squares regression models wherein 5-mCpG rates measured on each of the two haplotypes of each individual was the outcome variable and the fraction of mRNA expression originating from one of the two haplotypes of each individual was the main predictor with the addition of the following covariates: age of individuals at blood draw, sex (male, female), parent of origin (maternal, paternal), measurements of cell-type abundance (quantitative) along with the first five principal components derived from singular value decomposition analyses of methylation across the cohort, see section ‘Covariates.’

We tested mRNA isoforms if the TSS was located within 100 kb of the MDS. We tested MDSs where at least six sequences were available for estimating the 5-mCpG rates (Supplementary Fig. 2c), and we therefore apply the same criteria to mRNA isoforms. Further, we performed the regression if >100 observations were informative for the outcome, main predictor and covariates.

The same procedure was followed to identify associations between individual CpG units and mRNA isoforms.

### Analysis of causality between methylation and expression

We performed the Mendelian randomization–Steiger test^43^ to assess whether CpG methylation is more likely to affect mRNA expression or whether mRNA expression is more likely to affect CpG methylation using 1,369 ASM-QTL sequence variants associating with both as instruments. The Mendelian randomization–Steiger test involves comparing the correlation of *G* with CpG M (*ρ*_GM_) with their correlation with *E* (*ρ*_GE_). If more sequence variants correlate more strongly with CpG methylation the conclusion is that CpG methylation is more likely to causally affect expression rather than vice versa. CpG methylation and mRNA expression are both measured imprecisely, which will reduce their correlation with the sequence variants. The reliability of the result from Mendelian randomization–Steiger test is estimated by the R statistic^43^. We also assessed how relatively more imprecise the mRNA expression measurements needed to have been for us to erroneously observe the ASM-QTL variants associating more strongly with CpG methylation than they did with mRNA expression by finding the minimum value *r* such that *rρ*_GE_ is less than *ρ*_GM_ half the time.

### Dissecting the effects of methylation on expression

Let *G* denote the allele count of a sequence variant standardized to have mean 0 and variance 1, *M* denote methylation levels and *E* denote expression levels. Let us also assume the model that sequence variants affect methylation and expression through distinct mechanisms, and that methylation and expression are normally distributed given *G*, then:

$${M}{{|}}G{\mathscr{ \sim }}{\mathscr{N}}\left({\beta }_{{{\mathrm{GM}}}}G,{\sigma }_{{\mathrm{M}}}^{2}\right)$$

$${E}{{|}}G{\mathscr{ \sim }}{\mathscr{N}}\left({\beta }_{{{\mathrm{GE}}}}G,{\sigma }_{{\mathrm{E}}}^{2}\right)$$

Under this model we can calculate the expectation of expression given methylation:

$$\begin{array}{l}{\rm{E}}\left({E}|{{M}}\right)={\rm{E}}\left({\rm{E}}\left({E}|{{G,M}}\right)|{M}\right)={\rm{E}}\left({\rm{E}}\left({E}|{{G}}\right)|{M}\right)\\\qquad\qquad\quad\;={\rm{E}}\left({\beta }_{{\rm{GE}}}{G}|{M}\right)={\beta }_{{\rm{GE}}}{\rm{E}}\left({G}|{M}\right)=\frac{{\beta }_{{\rm{GM}}}{\beta }_{{\rm{GE}}}}{{\sigma }_{\rm{M}}^{2}+{\beta }_{\rm{M}}^{2}}{M}=\frac{{\beta }_{{\rm{GM}}}{\beta }_{{\rm{GE}}}}{{\rm{Var}}({M})}{M}\end{array}$$

This tells us that the expectation of the regression coefficient we get when regressing expression on methylation, *β*_ME_, is $\frac{{\beta }_{{\rm{GM}}}{\beta }_{{\rm{GE}}}}{{\rm{Var}}({M})}$, or equivalently that ${{\rm{Var}}({M})\beta }_{{\rm{ME}}}={\beta }_{{\rm{GM}}}{\beta }_{{\rm{GE}}}$.

If we observe methylation or expression with random normally distributed noise, then this identity is not affected.

If methylation affects expression or if the sequence variants affect methylation and expression through a common mechanism, for example, TF binding, then the effect of methylation on expression will be greater than predicted by the effects of the sequence variant on methylation and expression:

$${{\rm{Var}}({M})\beta }_{{\rm{ME}}}\ge {\beta }_{{\rm{GM}}}{\beta }_{{\rm{GE}}}$$

### Reporting summary

Further information on research design is available in the Nature Portfolio Reporting Summary linked to this article.

## Online content

Any methods, additional references, Nature Portfolio reporting summaries, source data, extended data, supplementary information, acknowledgements, peer review information; details of author contributions and competing interests; and statements of data and code availability are available at 10.1038/s41588-024-01851-2.

### Supplementary information

Supplementary Information Supplementary Notes 1.1– 1.5; legends for Extended Data Figs.; description of contents in Data files 1–5; Figs. 1–8; Table 1; and list of Source Data files for main figures and Extended Data Figs.

Reporting Summary

### Source data

Source Data Fig. 1 Data relevant to Fig. 1.

Source Data Fig. 2 Data relevant to Fig. 2.

Source Data Fig. 4 Data relevant to Fig. 4.

Source Data Extended Data Fig. 2 Data relevant to Extended Data Fig. 2.

Source Data Extended Data Fig. 3 Data relevant to Extended Data Fig. 3.

Source Data Extended Data Fig. 5 Data relevant to Extended Data Fig. 5.

Source Data Extended Data Fig. 7 Data relevant to Extended Data Fig. 7.

## Data Availability

ASM-QTL summary statistics are available upon request from our website (www.decode.com/summarydata/), and can be used without restrictions by clicking ‘Summary data’ for this article. The legend for the data files can be found in the Supplementary Information file, or by clicking ‘Read me file’ on our website. Nanopore whole-genome and RNA-seq data are not publicly available because of Icelandic state law. However, sequence variants identified in the Icelandic population using whole-genome sequencing have been deposited at the European Variant Archive under accession PRJEB15197. Data from the following publicly available databases were used in the study: GWAS Catalog (trait-associated sequence variants, all studies v.1.0.3): https://www.ebi.ac.uk/gwas/; GTEx project (*cis*-acting expression QTLs, bulk tissue): https://gtexportal.org/home/; eQTLGen (*cis*-acting expression QTLs, phase I): https://www.eqtlgen.org/index.html; Ensembl v.87 (transcript isoforms): https://www.ensembl.org/index.html; VEP (sequence variant annotations): https://www.ensembl.org/info/docs/tools/vep/index.html; NCBI reference genome assembly (DNA sequence): https://www.ncbi.nlm.nih.gov/; ENCODE ChIP–seq data (ENCSR072QBN, ENCSR254XTB, ENCSR267YXV, ENCSR437MHW, ENCSR586POT, ENCSR393SYU, ENCSR167JFX, ENCSR785YRL): https://www.encodeproject.org/; ENCODE cCRE data (candidate *cis*-regulatory elements, v.3): https://screen.encodeproject.org; Fantom5 project (enhancer RNA, CAGE-seq for UBERON:0000178): https://fantom.gsc.riken.jp/5/; AlleleDB (ASB): http://alleledb.gersteinlab.org/; LOFTEE (high-quality annotation for loss of function sequence variants): https://github.com/konradjk/loftee. Source data are provided with this paper.

## Code availability

DNA samples were analyzed with two versions of our pipeline, v.3 (5,761 R9 flowcells) and v.4 (3,145 R9 flowcells). The main difference between the pipelines is the version of the basecaller. In v.3 squiggle data from PromethION was basecalled using Guppy v.3.3.0 (https://github.com/nanoporetech/remora), 3,826 flowcells, using either the ‘flipflop’ or ‘hac’ model or v.3.2.2 (536 flowcells), v.3.6.0 (675 flowcells) and v.4.0.14 (724 flowcells) using the ‘hac’ model. In v.4, all data were basecalled using guppy v.5.0.11, using the ‘sup’ model (dna_r9.4.1_450bps_sup_prom.cfg). All 7,179 individuals basecalled with guppy had a minimum reference-genome-aligned sequencing coverage of at least ten times at the time of analysis. Basecalled reads were mapped to the human reference genome GRCh38 with minimap2 (https://github.com/lh3/minimap2), v.2.14-r883 (5,748 flowcells), v.2.17-r941 (13 flowcells) and v.2.22-r1105 (3,145 flowcells). The aligned reads were sorted using samtools sort and stored in a BAM file. Nanopolish v.0.11.0 and v.0.13.3 were used to detect CpG methylation in nanopore sequences. Data analysis was performed in R (v.3.6.0; https://www.r-project.org/), and the codes were deposited on our GitHub page (https://github.com/DecodeGenetics/) under the repository name: Stefanssonetal-Nature-Genetics-2024 (10.5281/zenodo.12103852)^64^.
