# Supplementary material for: The correlation between CpG methylation and gene expression is driven by sequence variants
Source: Nat Genet. 2024 Jul 24;56(8):1624–31. doi: 10.1038/s41588-024-01851-2 (PMC11319203; doi:10.1038/s41588-024-01851-2)
Supplement: Supplementary file 1 — Supplementary Notes 1.1– 1.5; legends for Extended Data Figs.; description of contents in Data files 1–5; Figs. 1–8; Table 1; and list of Source Data files for main figures and Extended Data Figs. [file 41588_2024_1851_MOESM1_ESM.pdf]

nature genetics

Supplementary information https://doi.org/10.1038/s41588-024-01851-2

The correlation between CpG methylation and gene expression is driven by sequence variants

In the format provided by the authors and unedited

Table of contents:

1. Supplementary Notes
2. Legends for Extended Data Figures
3. Legends for Data files
4. Supplementary Figures
5. Supplementary Tables
6. List of Source Data

Other supplementary materials for this manuscript include the following summary data which can be downloaded from our website (www.decode.com/summarydata/):

Data-S1.tab†: Summary of sequence variants associated with 5-mCpG rates of CpG units

Data-S2.tab†: Coordinates of MDSs

Data-S3.tab†: Summary of sequence variants associated with 5-mCpG rates of MDSs

Data-S4.tab†: Summary of MDSs associated with expression of mRNA isoforms

Data-S5.tab†: Summary of CpG units associated with expression of mRNA isoforms

* Corresponding authors. E-mails: Olafur.Stefansson@decode.is (O.A.S.); kstefans@decode.is (K.S.)

† Coordinates correspond to those of the 1-based hg38 (human reference genome GRCh38).

1. Supplementary Notes

1.1. Nanopore sequencing and oxBS performed in the same DNA samples

We performed a comparison between nanopore sequenced DNA samples isolated from 132 individuals that had previously been analysed by oxidative whole-genome bisulfite sequencing (oxBS)[32]. We showed that the average coverage of nanopore sequenced DNA samples is indicative of how consistent the 5-mCpG rates are with CpG methylation measured by oxBS (**Supplementary Figure 2A&B**). We apply this result in our study by excluding individuals whose DNA samples have been sequenced to less than 10x average coverage.

We binned each CpG according to the number of sequences used to compute its 5-mCpG rate in each sample analyzed by nanopore sequencing. Based on the Pearson's correlation coefficient we then determined how consistent the 5-mCpG rates in each such bin were to 5-mCpG rates measured with high coverage (>25 sequences for each CpG site) by oxBS (**Supplementary Figure 2C**). The results indicated that, for each DNA sample, the 5-mCpG rate was more reliably estimated when more than 9 sequences were used in the estimate.

For each CpG-unit, we calculated the average 5-mCpG rate across DNA samples from individuals measured by nanopore sequencing (n=7,179) and oxBS (n=132) to then compute the Pearson's correlation coefficient across averaged 5-mCpG rates. We refer to this correlation as per CpG average Pearson correlation (PCAPC). By looking at all CpGs detected by both methods, we found strong correlation i.e., PCAPC=0.9594 (95%CI:0.9594,0.9595).

We identified five different attributes of CpG units (see methods section entitled: **CpG methylation analysis by nanopore sequencing**) that negatively affected the PCAPC (**Supplementary Figure 3A**), and we removed those CpG units from the study (**Supplementary Figure 3B**).

1.2. Long range phasing of sequences detected by Nanopolish and oxBS

Phasing of the sequences is important for resolving heterogeneity in the data as phasing enables us to quantify 5-mCpG rates of CpG sites separately for each of the two parental chromosomes. For arrays such as HumanMethylation BeadChip developed by Illumina, phasing is not possible and phasing of short sequences from whole-genome bisulfite sequencing is also problematic as, due to the short lengths of sequenced DNA, it is less likely that the sequences contain a known sequence variant that is heterozygous in the individual. Commonly used long-read phasing tools include Margin (github.com/UCSC-nanopore-cgl/margin) and LongPhase. We used a different approach, as we have nearly complete parent of origin

information for our genotype data[56], we could assign each sequence to a parental haplotype based on agreement with the haplotypes (see methods section entitled: **Assignment of 5-mCpG status to parental haplotypes**).

Short-read sequencing reads were assigned to parental haplotypes by examining the phasing status of 17,649,586 sequence variants and one heterozygous variant needed per sequence[32].

7.6% of sequences detected by oxBS were successfully phased whereas 39.5% of sequences detected by nanopore sequencing were successfully phased. The main limitation for the long-read phasing is the set of heterozygous SNPs per individual, as 97.6% of the nanopore sequences covering at least 1 heterozygous SNP, were phased.

On average, 21.87% of the CpGs were phased for oxBS per sample, but 81.05% for nanopore sequencing.

Additionally, we found that 8.61% of aligned bases were phased for oxBS, but 71.20% for nanopore sequencing.

The phasing is affected by sequence length which explains the fact that despite applying stricter conditions on the phasing for long-read sequencing we were still able to phase a higher fraction of sequences and more CpGs on average per individuals than for short-reads. Average sequence length (N50) for phased sequences was 15,798 bp for nanopore sequencing, while average length for sequences that were not phased was 9,732 bp.

When working with phased data, the set of high-quality CpGs should be refined to exclude CpGs where the phasing is often impossible. We calculated the fraction of phased sequences out of all sequences covering SNPs that we are able to phase for each CpG and suggest excluding CpGs where the fraction of phased sequences is less than 0.3 as performed in this study (see methods section entitled: **CpG methylation analysis by nanopore sequencing**).

1.3. Replication analysis: GoDMC study

The GoDMC[40] study is currently the largest study on the effect of sequence variants on 5-mCpG although testing less than 450 thousand CpGs (*versus* 15.3 million CpG units measured in our study). 152,794 of the sequence variants identified as *cis*-methylation QTLs by GoDMC were mapped to our high-quality set of ~34.4 million sequence variants according to chromosome position and alleles. Note, in line with our analysis, we restricted to GoDMC sequence variants located within 100kb of the affected CpG. 112,193 out of the 152,794 (73.4%) sequence variants identified as *cis*-methylation QTLs by GoDMC were in high LD ($r2>0.80$) to those identified in our study in association with 5-mCpG rates of individual CpG units.

To determine whether the observed overlap with GoDMC sequence variants is higher than expected by chance alone, we binned the ~1 million 5-mCpG associated sequence variants identified in our study, hereafter referred to as the observed sequence variants, into seven MAF bins (0-0.01,>0.01-0.05,>0.05-0.1,>0.1-0.2,>0.2-0.3,>0.3-0.4,>0.4-0.5). We counted the number of observed sequence variants in each MAF bin and sampled the same number of sequence variants from the 34.4 million sequence variants (located within 100kb from each of the 15.3 million measured CpG units). Hence, we obtained ~1 million sampled sequence variants matched to the observed sequence variants with respect to MAF. We found that approximately 24.4% of the ~1 million sampled sequence variants were in high LD to at least one sequence variant identified by GoDMC as a *cis*-methylation QTL. The 73.4% observed overlap with our findings is significantly higher than the 24.4% expected overlap (Binomial test, $P < 0.05$).

Further, we tested the GoDMC *cis*-methylation QTLs in our cohort using the same sequence variants and CpG positions as reported by GoDMC. The effect sizes observed in our cohort were consistent with those reported by GoDMC (Pearson's r =0.736, 95%CI:0.734,0.739).

We note, however, that the effect sizes in our cohort tended to be lower than those reported by GoDMC; the regression effect on GoDMC effect sizes for deCODE effect sizes was $\hat{\beta}$=1.57 (97%CI:1.56,1.58). 5-mCpG rates measured using >12 sequences were highly consistent with rates measured by bisulfite sequencing (Supplementary Figure 2C). By restricting to haplotypes where >12 sequences were available for measuring the 5-mCpG rate we demonstrated that both the coverage/read depth of the CpG unit and the number of observations, i.e. the number of haplotypes measured with >12x coverage/read depth, were likely important contributors to the observed bias towards lower effect sizes in our cohort (**Supplementary Figure 7**).

1.4. Enrichment analysis: ASM-QTLs stratified by TSS proximity

We find that 38.4% (38.0,38.7%) of ASM-QTLs (i.e., 30,889 out of 80,503) reside within 25kb from the TSS of a protein coding gene. 53.1% (95%CI:52.4,53.8%) of GWA signals reside within 25kb from the TSS of a protein coding gene. Hence, in comparison to index variants of GWA signals, the index variants of ASM-QTLs are less frequently found near TSSs (<25kb).

We therefore sought to explore the question of whether proximity to protein coding TSSs influences the enrichment of ASM-QTLs among GWA signals.

To this end we binned sequence variants (other than missense and loss of function) according to distance to the nearest TSS as follows: >25kb (distal), 10-25kb, 5-10kb, <5kb. We then determined the enrichment of these non-coding sequence variants depending on whether or not they are ASM-QTLs.

We found that ASM-QTLs located <5kb from protein coding TSSs were approximately 70-fold (95%CI:45.8,96.4) enriched among GWA signals (**Supplementary Figure 8**), whereas other non-coding sequence variants located <5kb from protein coding TSSs were less enriched i.e., 5.5-fold (95%CI:4.8,6.2). Hence, although the 70-fold enrichment for ASM-QTLs among GWA signals seems impressive, there is already this 5.5-fold enrichment given the TSS proximity (<5kb from a protein coding TSS).

Note, in this model, non-coding sequence variants located >25kb from a protein coding TSSs were used as the baseline annotation (neutral enrichment). The corresponding category of ASM-QTLs, i.e. those located >25kb from protein coding TSSs, were enriched by 19.3-fold (95%CI:13.3,26) among GWA signals (**Supplementary Figure 8**). This estimate is similar to the 23.2-fold enrichment of ASM-QTLs among GWA signals that we computed in a model without considering TSS proximity (**Extended Data Figure 7**).

Hence, we show here that non-coding sequence variants located close (<5kb) to protein coding TSSs are enriched among GWA signals. ASM-QTLs, however, are far more enriched among GWA signals regardless of whether or not they are located close to protein coding TSSs.

1.5. Summary of main limitations and strengths of 5-mCpG detection by nanopore sequencing

Currently, the main **limitations** of using nanopore sequencing for 5-mCpG detection are as follows: 1) sequencing coverage in the DNA sample as low coverage is indicative of unreliable measurements of 5-mCpG rates in the sample. 2) The presence of a sequence variant near or within a CpG site is problematic as this may cause erroneous 5-mCpG detection by some algorithms, like Nanopolish. 3) The observation of strand bias in 5-mCpG detection at a CpG site is fairly frequent and we demonstrated that this is indicative of erroneous 5-mCpG detection.

The main **strengths** are as follows: 1) Nanopore sequencing delivers longer sequences than other sequencing methods, including whole genome bisulfite sequencing. This property of nanopore sequencing considerably enhances the efficiency of phasing the sequences. Phasing is important for resolving the heterogenity in the measurements of 5-mCpG rates, a task that cannot be accomplished with array-based methods. 2) The large number of CpG sites detected by nanopore sequencing allows us to access regions in the genome that are not accessible using array-based methods, thereby opening avenues for making novel discoveries.

In our previous work[64] we compared, in more detail, different algorithms and long read sequencing technologies for detecting 5-mCpG status in DNA samples. We note that Remora (of Guppy) exhibits less sensitivity to sequence variation in close proximity to the CpG site thereby enabling high-quality measurements of more CpGs than can be reliably measured by Nanopolish.

2. Legends for Extended Data Figures

Extended Data Figure 1: **Methylation depleted sequences**. Lines on the y-axis represent unmethylated haplotypes found in different individuals that overlap in position, with chromosomal position on the x axis. (a) For each such “cluster of overlapping unmethylated haplotypes”, as shown here, we look for the most frequently occurring unmethylated haplotype, shown in red. This “dominant” form is then taken as the representative for the cluster of unmethylated haplotypes, which then enables comparison in 5-mCpG haplotype rates over the same coordinates (those of the representative) across all individuals in the cohort. (b) An example cluster where two (or more) representatives were found which we identify, and measure separately as distinct entities.

Extended Data Figure 2: **Validation of ASM-QTL influences in oxBS-seq data**. ASM-QTLs validated using oxBS in independent DNA samples derived from whole blood of forty-five individuals. Note, these forty-five individuals were not included in the larger cohort of 7,179 nanopore sequenced individuals. The effect size of each ASM-QTL on the 5-mCpG haplotype rates of MDSs in oxBS sequenced individuals, y-axis, are plotted against the effect size of the same ASM-QTLs on 5-mCpG haplotype rates of the corresponding MDS in nanopore sequenced samples, x-axis. We binned the validation tests according to the number of haplotypes (n) that were available for validation testing in oxBS individuals as indicated on the top of each figure. A least squares regression line, where the effect sizes in the oxBS validation cohort are used as the outcome and those in nanopore sequenced cohort as the predictor, is shown in each figure, blue color, along with a diagonal line of identity, red color, as we expect the regression coefficient to be equal to one (and an intercept of zero) if the effect sizes are identical in the oxBS and nanopore sequenced individuals. The regression coefficients and their 95% confidence intervals are shown at the top left-hand corner of each figure. Note that as the number of informative haplotypes (n) in the oxBS validation cohort increases, the regression coefficient approaches that of a diagonal line indicating that the effect sizes become more similar as the number of haplotypes available for testing (n) in the oxBS cohort increases.

Extended Data Figure 3: **MDSs associated with gene expression.** Distance, x-axis, from MDS midpoints to the TSS locations of the associated mRNA transcript isoforms *versus* the effect size on expression, y-axis. MDS midpoints are represented as black dots, and the MDSs are represented as ranges i.e., horizontal lines. (a) Distal associations i.e., where the MDS does not contain the TSS of the associated mRNA. (b) Promoter associations i.e., where the MDS contains the TSS of the associated mRNA, where the MDS is represented by a line (left to right) indicating the position relative to the TSS of the associated mRNA. (a) and (b) TSS is represented as the origin of the x-axis (x=0), shown as a vertical dashed line, red. Note, we modify the sign of the distance such that negative distances reflect upstream positions of the MDS midpoint relative to the TSS of the associated mRNA. Hence, the upstream positions indicate that the MDS midpoint does not intersect with the direction of mRNA transcription. In contrast, downstream positions indicate that the MDS midpoint does intersect with the direction of mRNA transcription.

Extended Data Figure 4: Functional attributes of ASM-QTLs. ASM-QTLs were more likely than expected by chance, to be found in high LD (r^2 >0.80) to A) sequence variants that influence the DNA binding affinity to transcription factors SPI1, CTCF and EBF1, and cohesin protein STAG1 and B) sequence variants located within regulatory sequences defined by ENCODE[33], i.e., candidate cis-regulatory elements (*version 3*). Note, sequence variants that influence ASBs were identified in Chen et al[42]. The *P*-values shown, unadjusted for multiple comparison, were computed using our permutation-based method described under methods section „ASM-QTL in functional annotation maps“.

Extended Data Figure 5: ASM-QTLs dominate in correlations between MDSs and mRNA. (**a**) Example of an association found between expression of VAMP5-201 (ENST00000306384), y-axis, and 5-mCpG haplotype rates, x-axis, of an MDS (chr2:85584168-85584976) located within the CpG island promoter of the TSS; i.e., an example of CpG island promoter methylation. (**b**) The fraction of variance in VAMP5-201 mRNA isoform expression explained by (*top)* ASM-QTL found in association with the MDS *(middle)* CpG methylation of the MDS and *(bottom)* CpG methylation of the MDS after correction for the ASM-QTL. This same association between MDS at chr2:85584168-85584976 and mRNA expression of VAMP5-201 is then shown separately on (**c**) the reference- and (**d**) the alternative alleles of ASM-QTL chr2:85580659:IG.0:0, respectively. Least squares regression line is shown within each of three scatter plots, in blue color, and the estimated regression coefficient and their 95% confidence intervals are shown at the bottom, along with the corresponding two-sided *P*-values.

Extended Data Figure 6: Modeling the impact of ASM-QTL on methylation and expression. Model diagrams (leftmost column) describing the four different hypotheses on the mechanism by which an ASM-QTL genotype (G) influences CpG methylation (M) and gene expression (E). The outcomes of two different tests (MR-Steiger and our method of comparing $\hat{\beta}_{GM}\hat{\beta}_{GE}$ and $\hat{\sigma}_M^2\hat{\beta}_{ME}$) are shown; "v" shape indicates that the model is supported by the test, whereas "x" indicates that the model is not supported by the test and empty cells indicates that the test is unable to consider whether or not the model is supported. For clarity, each model is labelled with a number which are then used as reference to each diagram in the main text and in **Figure 3**.

Extended Data Figure 7: Varied contribution of sequence variants to human trait variability. Enrichment, x-axis, of ASM-QTLs and other sequence variant annotations, y-axis, among GWA signals found in various human diseases and other traits. The solid points (black) represent the measure of center, i.e., the enrichment point estimates and the horizontal lines (black) represent their 95%CIs. The number of sequence variants in each annotation is shown within parentheses on the y-axis. Shown are the enrichment point estimates, points, and their 95% confidence intervals, horizontal lines, for each sequence variant annotation, and the point of neutral enrichment on the x-axis as vertical line, blue. UTR=Untranslated Region, DHS=DNase hypersensitivity site, MDSs=Methylation depleted sequences.

3. Legends for Data files

Data files can be downloaded from our website: www.decode.com/summarydata/

Data-S1.tab: Sequence variants associated with 5-mCpG rates of individual CpG units; GRCh38 coordinates, 1-based.

Chrom*: The chromosome of sequence variants and CpG units*
SeqVariant_start*: The nucleotide starting position of the sequence variant*
SeqVariant _end*: The nucleotide ending position of the sequence variant*
SeqVariant _name*: The inhouse name of the sequence variant*
SeqVariant _rsname*: The rs-identifier of the sequence variant*
SeqVariant _ref*: The reference allele of the sequence variant*
SeqVariant _alt*: The alternative allele of the sequence variant*
SeqVariant _minorallele*: The minor allele of the sequence variant*
SeqVariant _MAF*: The minor allele frequency of the sequence variant*
SeqVariant _LD_count*: The number of sequence variants found in high LD (r^2>0.8) to the sequence variant*
SeqVariant _vartype*: sequence variant type (SNV=single nucleotide variant, Indel, Micosatellite and SV=Structural variant)*
SeqVariant _rank*: The rank of the sequence variant: primary=the most significantly associated variant, secondary= the most significantly associated variant on the major allele of the primary*
CpG_start*: The nucleotide starting position of the CpG unit*
CpG_end*: The nucleotide ending position of the CpG unit*
CpG_ref_methylrate*: The average 5-mCpG rate of the CpG unit on reference alleles*
CpG_alt_methylrate*: The average 5-mCpG rate of the CpG unit on alternative alleles*
SeqVariant _5mCpG_effectsize*: The effect size (SD units) of the sequence variant on 5-mCpG rates of the CpG umit*
SeqVariant _5mCpG_95CI*: The 95%CI of the sequence variant effect size*
n_haplotypes*: The number of informative haplotypes used to identify the sequence variant*
GWAS_disease*: Comma separated list of disesases (or disorders) found in association to a sequence variants in high LD (r^2>0.8) to the 5-mCpG-associated sequence variant*
GWAS_disease_markers*: Comma separate list of disease-associated sequence variants in high LD (r2>0.8) to the 5-mCpG-associated sequence variant. Format: disease-associated variant name (disease-associated variant effect size | r^2 of disease-associated variant to 5-mCpG-associated sequence variant), comma separated list in the same order as „GWAS_disease" column*
GWAS_other.traits*: Similar to „GWAS_disease_markers" columnt except for traits not classified as either disease or disorder*
GWAS_other.traits_markers*: Similar to „GWAS_disease_markers" except for traits not classified as either disease or disorder, comma separated list in the same order as „GWAS_other.traits" column*

Data-S2.tab: The coordinates of the methylation depleted sequences (MDSs); GRCh38 coordinates, 1-based.

Chrom*:The chromosome of the MDS*
Start*:The nucleotide starting position of the MDS*
End*:The nucleotide ending position of the MDS*
MDS*:Coordinate-identifier of the MDS*
Class*:The type of MDS*

Data-S3.tab: Sequence variants associated with 5-mCpG rates of MDSs; GRCh38 coordinates, 1-based.

Chrom*: The chromosome of sequence variants and MDSs*
SeqVariant_start*: The nucleotide starting position of the sequence variant*
SeqVariant _end*: The nucleotide ending position of the sequence variant*
SeqVariant _name*: The inhouse name of the sequence variant*
SeqVariant _rsname*: The rs-identifier of the sequence variant*
SeqVariant _ref*: The reference allele of the sequence variant*
SeqVariant _alt*: The alternative allele of the sequence variant*
SeqVariant _minorallele*: The minor allele of the sequence variant*
SeqVariant _MAF*: The minor allele frequency of the sequence variant*
SeqVariant _LD_count*: The number of sequence variants found in high LD (r^2>0.8) to the sequence variant*
SeqVariant _vartype*: sequence variant type (SNV=single nucleotide variant, Indel, Micosatellite and SV=Structural variant)*
SeqVariant _rank*: The rank of the sequence variant: primary=the most significantly associated variant, secondary= the most significantly associated variant on the major allele of the primary*
MDS_start*: The nucleotide starting position of the MDS*
MDS_end*: The nucleotide ending position of the MDS*
MDS_ref_methylrate*: The average 5-mCpG rate of the MDS on reference alleles*
MDS_alt_methylrate*: The average 5-mCpG rate of the MDS on alternative alleles*
SeqVariant _5mCpG_effectsize*: The effect size (SD units) of the sequence variant on 5-mCpG rates of the MDS*

SeqVariant _5mCpG_95CI: *The 95%CI of the sequence variant effect size*
n_haplotypes: *The number of informative haplotypes used to identify the sequence variant*
GWAS_disease: *Comma separated list of disesases (or disorders) found in association to a sequence variants in high LD (r^2>0.8) to the MDS-associated sequence variant*
GWAS_disease_markers: *Comma separate list of disease-associated sequence variants in high LD (r2>0.8) to the MDS-associated sequence variant. Format: disease-associated variant name (disease-associated variant effect size | r^2 of disease-associated variant to MDS-associated sequence variant), comma separated list in the same order as „GWAS_disease“ column*
GWAS_other.traits: *Similar to „GWAS_disease_markers“ columnt except for traits not classified as either disease or disorder*
GWAS_other.traits_markers: *Similar to „GWAS_disease_markers“ except for traits not classified as either disease or disorder, comma separated list in the same order as „GWAS_other.traits“ column*

Data-S4.tab: 5-mCpG rates of MDSs found in association with expression of mRNA isoform; GRCh38 coordinates, 1-based.

Chrom: *The chromosome of the MDS*
Start: *The nucleotide starting position of the MDS*
End: *The nucleotide ending position of the MDS*
MDS: *Coordinate-identifier of the MDS*
mRNA_isoform: *Ensembl identifier of the mRNA isoform*
Gene: *The gene identifier of the mRNA isoform*
effectsize: *The effect size (SD units) of the 5-mCpG rate of the MDS on expression level of the mRNA isoform*
p_value: *P-value for the MDS in the association between 5-mCpG rates and mRNA isoform expression*

Data-S5.tab: 5-mCpG rates of CpG units found in association with expression of mRNA isoform; GRCh38 coordinates, 1-based.

Chrom: *The chromosome of the CpG unit*
Start: *The nucleotide starting position of the CpG unit*
End: *The nucleotide ending position of the CpG unit*
CpG: *Coordinate-identifier of the CpG unit*
mRNA_isoform: *Ensembl identifier of the mRNA isoform*
Gene: *The gene identifier of the mRNA isoform*
effectsize: *The effect size (SD units) of the 5-mCpG rate of the CpG unit on expression level of the mRNA isoform*
p_value: *P-value for the CpG unit in the association between 5-mCpG rates and mRNA isoform expression*

---

4. Supplementary Figures

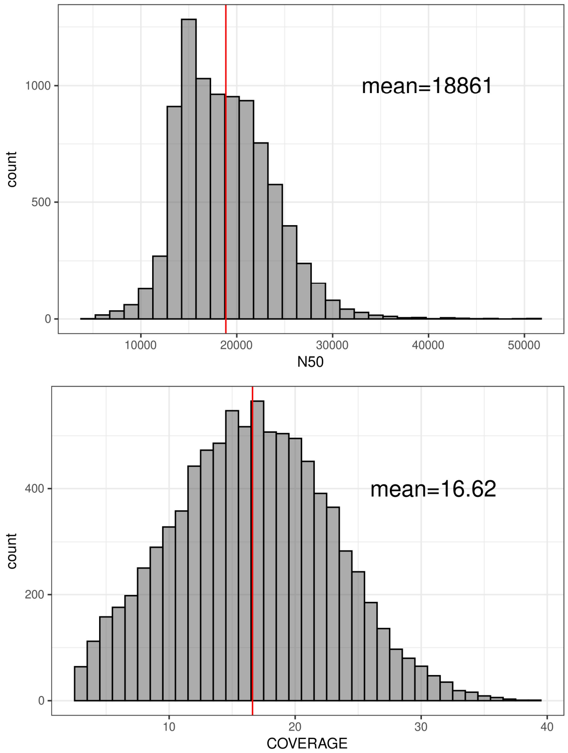

Supplementary Figure 1: Shown are histograms for N50, upper panel, and the average coverage for each DNA sample, lower panel.

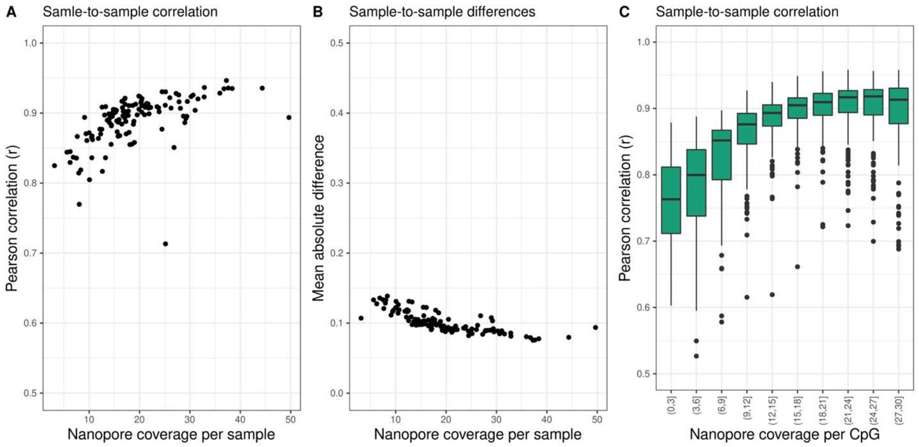

Supplementary Figure 2: Nanopore sequencing and oxBS performed in the same DNA samples. The consistency in 5-mCpG rates measured by nanopore sequencing and oxBS in DNA samples isolated from the same 132 individuals was estimated by (A) Pearsons r correlation coefficient, y-axis, and (B) mean of the absolute differences in 5-mCpG rates of each CpG site, y-axis, with respect to nanopore sequencing coverage in each sample on the x-axis. Note, the y-axes in A and B are limited to enhance readability of the data. (C) CpG coverage underlying the 5-mCpG rates, i.e., the number of sequences that were used to compute the 5-mCpG rate for a given CpG, in the nanopore sequenced samples, x-axis, influences the consistency (Pearsons r), y-axis, with 5-mCpG rates of CpGs measured with high coverage (>25 sequences per CpG) by oxBS. The center line (solid black) shown in each box represents the median; box limits represent upper and lower quartiles; vertical lines represent the range spanning 1.5x quartiles. Note, the y-axis is limited (0.5, 1). Nanopore sequencing and oxBS performed in the same DNA samples.

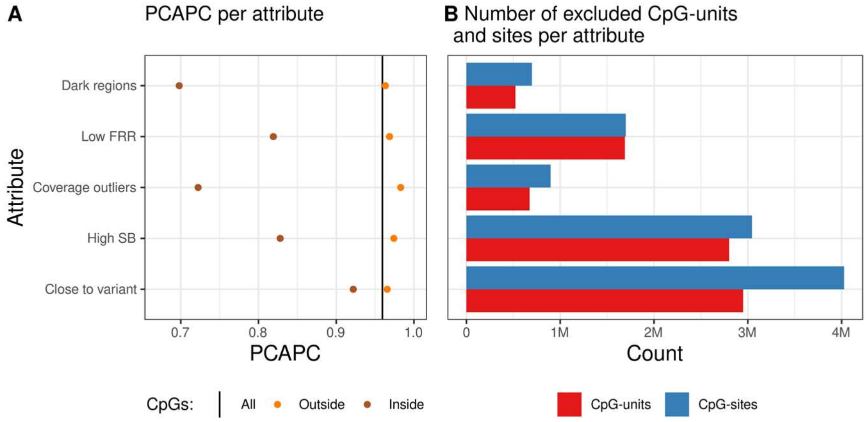

Supplementary Figure 3: The quality of 5-mCpG rate measurements varies by different attributes of the sequenced DNA. (A) Shown are PCAPC estimates, x-axis, for CpG sites located outside (orange colour) and inside (brown colour) of different attributes of the sequenced DNA, y-axis, and the PCAPC estimate based on all CpGs (vertical line, black colour). (B) The number of CpG-units (red) and CpG-sites (blue), x-axis, found inside of each attribute, y-axis. Attributes on y-axis are as follows (top to bottom): „Dark regions“ contain sequence repeats that cause mapping to be unreliable, „Low FRR“ (Fraction of Reliable Reads ≤0.5) is indicative of problematic sequences, „Coverage outliers“ (defined where the average is <0.5 or >1.5) are indicative of repetitive regions, „high SB“ (Strand Bias ≥0.20) is a measure of strand bias in 5-mCpG detection based on the absolute difference between methylation ratio on forward and reverse strand, „Close to variant“ indicates the presence of a known SNP locus (MAF>0.001) within 5bp of measured CpGs.

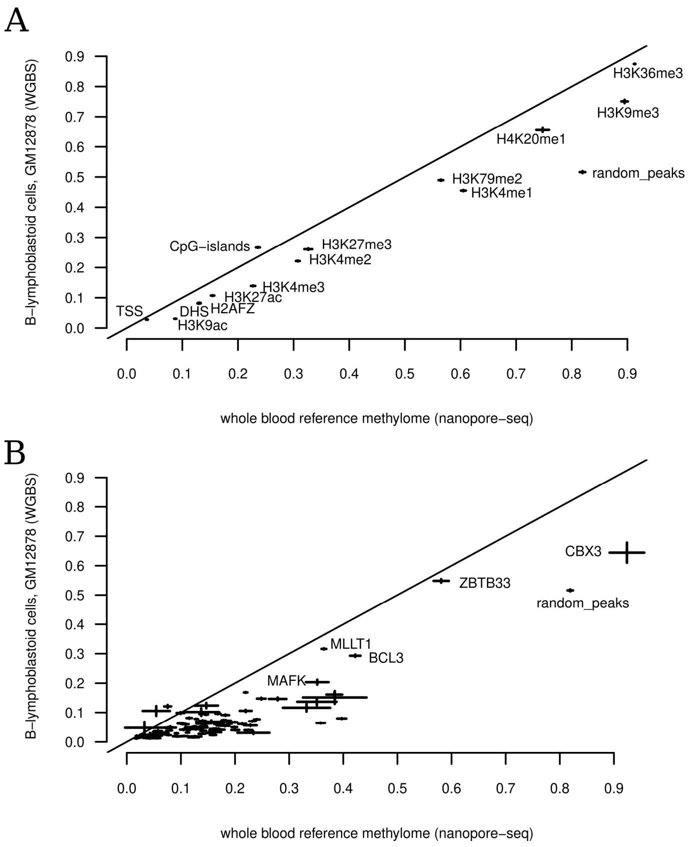

Supplementary Figure 4: Depletion of 5-mCpGs in functional regions. (A) Genomic attributes defined in the well-studied B-lymphoblastoid GM12878 cell line, i.e. regions marked by histone modifications (ChIP-seq) along with transcriptional start sites (RAMPAGE assay) and CpG islands. 5-mCpG measured by WGBS in GM12878 (Encode project), y-axis, and measures of 5-mCpG by nanopore sequencing of whole blood samples, x-axis. (B) TF protein binding sites defined in B-lymphoblastoid GM12878 cell line have similar 5-mCpG analyzed by WGBS in GM12878, y-axis, as those measured by nanopore sequencing in whole blood samples, x-axis. CBX3 is also known as HP1-γ (heterochromatin protein 1 homolog gamma). In both (A) and (B), we show the means of 5-mCpG rates of the inner core region (-50 to +50bp relative to midpoint) and 95%CI for each genomic attribute defined in GM12878; i.e. measures of mean methylation within the same region coordinates for GM12878 (WGBS), y-axis, and our whole-blood samples (nanopore sequencing), x-axis. Additionally, a diagonal line (y=x) is drawn in both (A) and (B). 50 thousand „random_peaks" were selected from randomly selected locations in the genome shown on the figure as genome-wide reference points. A trend towards overall lower 5-mCpG in B-lymphoblastoid GM12878 cells is a well-known feature of methylomes in cultured cell lines. Abbreviations: DHS=DNase hypersensitivity sites, TSS=Transcription start sites.

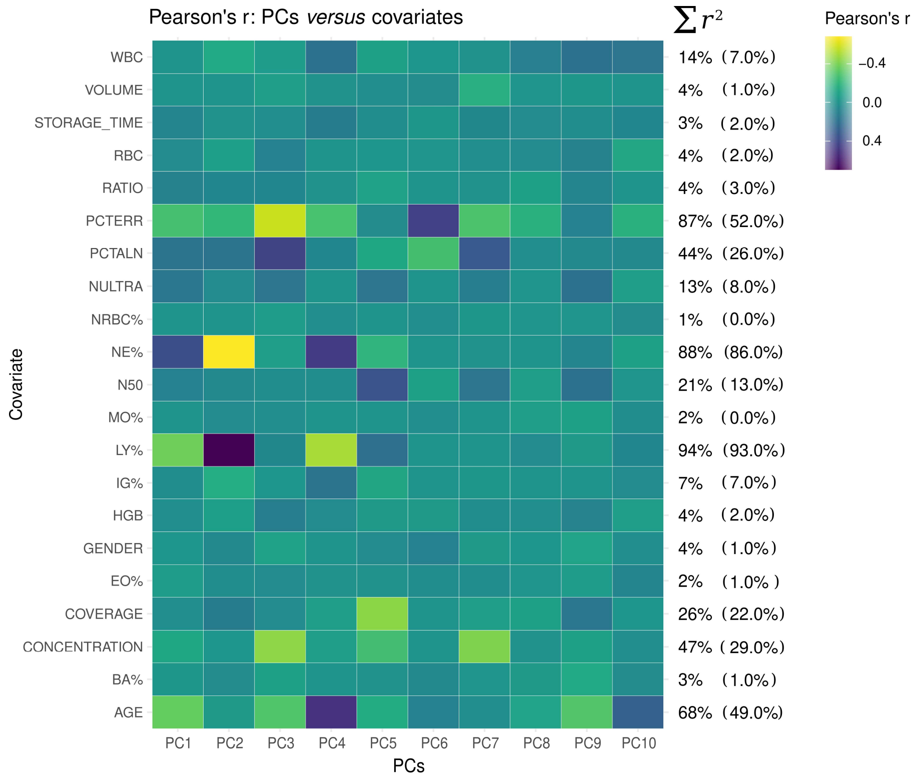

Supplementary Figure 5: Pearson´s correlation coefficient between PCs and covariates. The sum of r^2 for each covariate (y-axis) across PCs 1 to 10 are shown on the right-hand side of the heatmap and, within parentheses, the sum across PCs 1 to 5. The correlations were computed for each covariate against each of the ten PCs; here, using the PCs computed from a random sample of CpG units (hq) located on autosomes except for chromsome 1. Abbreviations: WBC=White blood cells, RBC=Red blood cells, PCTERR=Percent error, PCTALN=Percent alignment, NULTRA=Number of ultra long sequences, NRBC=Fraction of nucleated red blood cells, NE=Neutrophils, MO=Monocytes, LY=Lymphocytes, IG=Immature granulocytes, HGB=Hemoglobin, EO=Eosinophils, BA=Basophils.

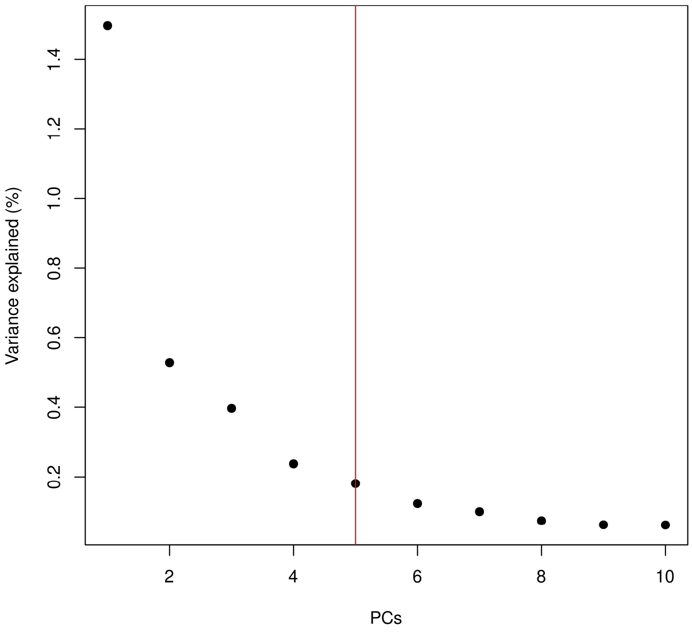

Supplementary Figure 6: Percent of the variance in 5-mCpG rates explained by each of the first ten PCs. The first five PCs, vertical line (red), explain ~2.84% of the variance and each of the other PCs (i.e. the sixth, seventh etc.) explain less than 0.12%. The PCs were computed using randomly sampled autosomal CpG units (hq). PC=Principal component.

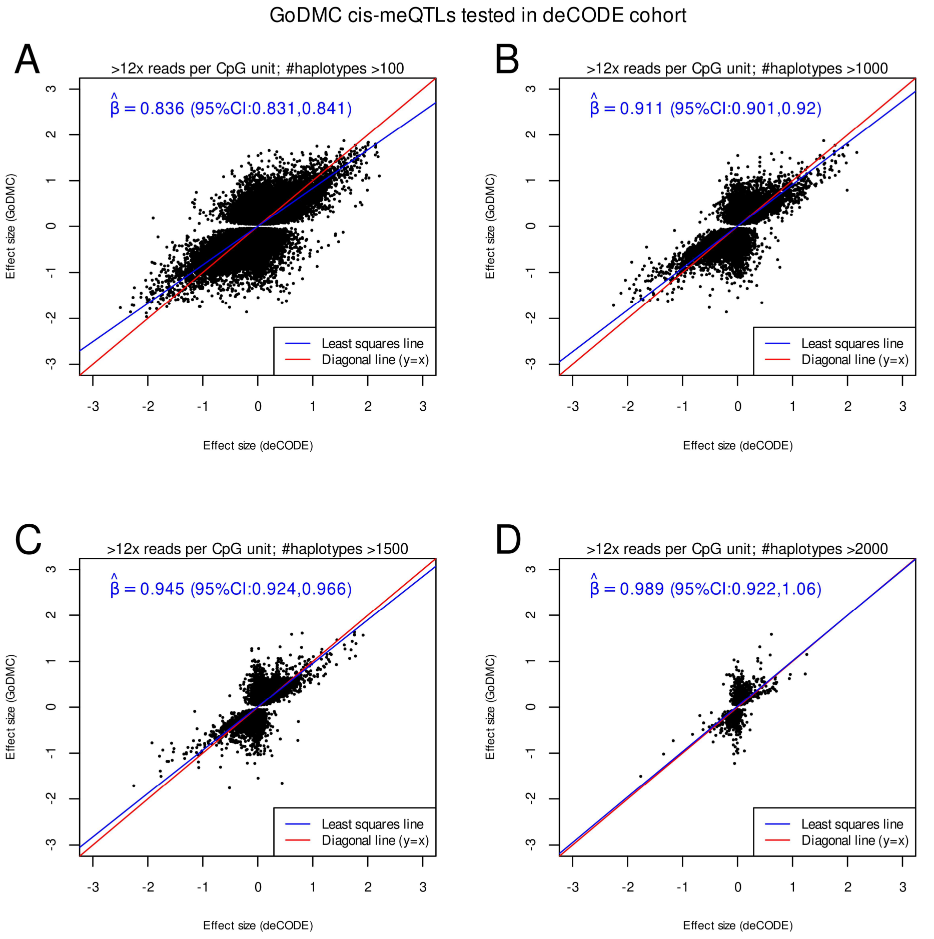

Supplementary Figure 7: GoDMC *cis*-methylation QTLs tested for association in deCODE cohort after restricting to haplotypes with at least 12x coverage/read depth for measuring the 5-mCpG rate. The effect size (SD units) of sequence variants in the GoDMC study, y-axis, plotted against the effect size (SD units) of the same sequence variants on the same CpGs in our cohort, x-axis. The number of observations, i.e. the number of haplotypes with >12x reads (based on Supplementary Figure 2C), that were available to test for associations between a *cis*-meQTL (GoDMC) sequence variant and CpG unit methylation in our cohort is shown at the top of each figure as follows: (A) >100 haplotypes (107,046 *cis*-meQTLs), (B) >1000 haplotypes (30,332 *cis*-meQTLs), (C) >1500 haplotypes (9,807 *cis*-meQTLs) and (D) >2000 haplotypes (1,661 *cis*-meQTLs). The regression coefficient on GoDMC effect sizes, y-axis, for deCODE effect sizes, x-axis, is shown in blue colour at the top-left of each figure. The least squares regression line (blue) and diagonal line (red) are shown.

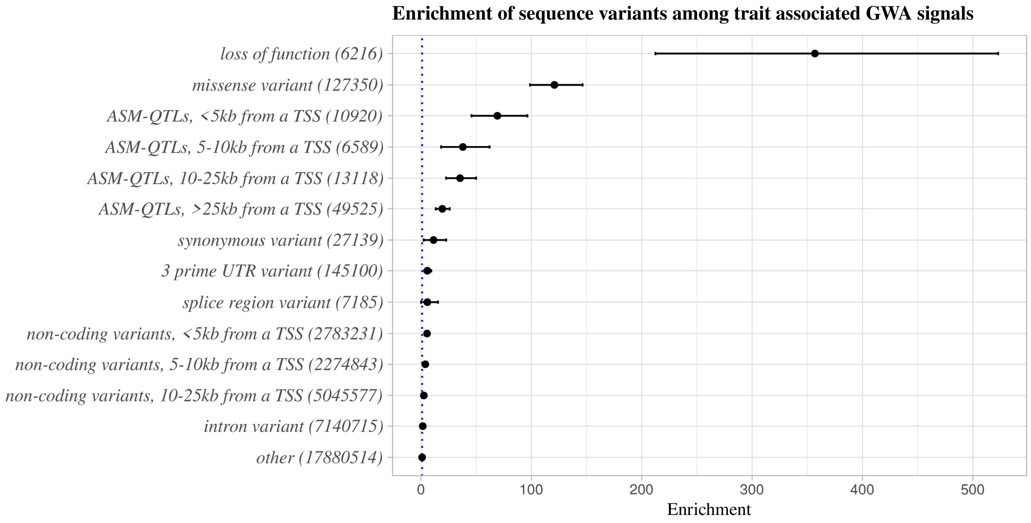

Supplementary Figure 8: The enrichment of ASM-QTLs among GWA signals varies according to distance from protein coding TSSs. The enrichment, x-axis, of each ASM-QTLs category among GWA signals found in various human diseases and other traits relative to other sequence variant annotations, x-axis. The solid points (black) represent the measure of center, i.e., the enrichment point estimates and the horizontal lines (black) represent their 95%CIs. The number of sequence variants in each annotation is shown within parentheses on the y-axis. Shown are the enrichment point estimates, points, and their 95% confidence intervals, horizontal lines, for each sequence variant annotation, and the point of neutral enrichment on the x-axis as vertical line, blue. TSS=Transcription Start Site.

5. Supplementary Tables

Supplementary Table 1: Annotations of sequence variants used in the analysis of enrichment among trait associated sequence variants.

Index	Name	Merged from	Annotation description
1	missense_variant		*Variant resulting in substitution of an amino acid in the protein product of a gene.*
2	synonymous_variant		*Variant that does not affect the amino acid sequence of the protein product.*
3	splice_region_variant		*Variant located in region of splice site, 1-3 bases into the exon or 3-8 bases into the intron.*
4	3_prime_UTR_variant		*Variant located in the untranslated region of the 3´end of a gene.*
5	5_prime_UTR_variant		*Variant located in the untranslated region of the 5´end of a gene.*
6	DHS footprints, hematopoietic cells		*DHS footprints defined in hematopoietic cell types, defined by Vierstra et al*[6]
7	DHS footprints, non-hematopoietic cells		*DHS footprints defined in other, non-hematopoietic cell types, defined by Vierstra et al*[6]
8	intron_variant		*Variant located in an intron of a gene.*
9	loss_of_function	frameshift, stop gained, start lost, splice_donor_variant, splice_acceptor_variant	*Variant predicted to have loss of function consequences to a gene with high confidence according to loftee*[61]
10	ASM-QTL		*Alleles of sequence variants found in association with changes in 5-mCpG rates of MDSs on the same haplotype (defined in methods section entitled:* ***Allele-specific methylation quantitative trait loci)****.*
11	Methyl-depleted sequences, MDSs		*Variants located within DNA sequences depleted of 5-mCpGs; MDSs were defined as described in methods section entitled:* ***MDSs (methylation depleted sequences)****.*
12	Other	Merged from multiple other VEP annotations, but not those from 1 to 11.	*Variant that does not belong in any of the categories from 1 to 11.*

6. List of Source Data

6.1. Source data files for the main figures

Figure 1: source-data-fig1.xlsx

Figure 2: source-data-fig2.xlsx

Figure 3: Not applicable.

Figure 4: source-data-fig4.xlsx

6.2. Source data files for extended data figures

Extended Data Figure 1: Not applicable.

Extended Data Figure 2: source-data-EDFig2.xlsx

Extended Data Figure 3: source-data-EDFig3.xlsx

Extended Data Figure 4: Not applicable.

Extended Data Figure 5: source-data-EDFig5.xlsx

Extended Data Figure 6: Not applicable.

Extended Data Figure 7: source-data-EDFig7.xlsx
