# Supplementary material for: The correlation between CpG methylation and gene expression is driven by sequence variants
Source: Nat Genet. 2024 Jul 24;56(8):1624–31. doi: 10.1038/s41588-024-01851-2 (PMC11319203; doi:10.1038/s41588-024-01851-2)
Supplement: Supplementary file 2 — Reporting Summary [file 41588_2024_1851_MOESM2_ESM.pdf]

nature portfolio

Corresponding author(s): Olafur Andri Stefansson
Kari Stefansson

Last updated by author(s): Apr 19, 2024

Reporting Summary

Nature Portfolio wishes to improve the reproducibility of the work that we publish. This form provides structure for consistency and transparency in reporting. For further information on Nature Portfolio policies, see our Editorial Policies and the Editorial Policy Checklist.

Statistics

For all statistical analyses, confirm that the following items are present in the figure legend, table legend, main text, or Methods section.

n/a	Confirmed	
☐	☒	The exact sample size (*n*) for each experimental group/condition, given as a discrete number and unit of measurement
☐	☒	A statement on whether measurements were taken from distinct samples or whether the same sample was measured repeatedly
☐	☒	The statistical test(s) used AND whether they are one- or two-sided<br>*Only common tests should be described solely by name; describe more complex techniques in the Methods section.*
☐	☒	A description of all covariates tested
☐	☒	A description of any assumptions or corrections, such as tests of normality and adjustment for multiple comparisons
☐	☒	A full description of the statistical parameters including central tendency (e.g. means) or other basic estimates (e.g. regression coefficient) AND variation (e.g. standard deviation) or associated estimates of uncertainty (e.g. confidence intervals)
☐	☒	For null hypothesis testing, the test statistic (e.g. *F*, *t*, *r*) with confidence intervals, effect sizes, degrees of freedom and *P* value noted<br>*Give P values as exact values whenever suitable.*
☐	☒	For Bayesian analysis, information on the choice of priors and Markov chain Monte Carlo settings
☐	☒	For hierarchical and complex designs, identification of the appropriate level for tests and full reporting of outcomes
☐	☒	Estimates of effect sizes (e.g. Cohen's *d*, Pearson's *r*), indicating how they were calculated

Our web collection on statistics for biologists contains articles on many of the points above.

Software and code

Policy information about availability of computer code

Data collection	No software was used for data collection.
Data analysis	Description of the code used for Illumina short-read sequencing, alignment and imputation setup is described in the Data Descriptor "Whole genome characterization of sequence diversity of 15,220 Icelanders" by Jonsson et al, Scientific Data 2017.<br><br>Nanopore long-read sequencing was performed using PromethION machines and software used for the data analysis included Nanopolish (Simpson et al, Nat Methods 2017), Guppy, and Minimap2 (Li et al, Bioinformatics 2018) as described in methods section "CpG methylation analysis by nanopore sequencing".<br><br>DNA samples were analysed with two versions of our pipeline, v3 (5761 R9 flowcells) and v4 (3145 R9 flowcells). The main difference between the pipelines is the version of the basecaller. In v3 squiggle data from PromethION was basecalled using Guppy 3.3.0 (3826 flowcells) using either the 'flipflop' or 'hac' model or 3.2.2 (536 flowcells), 3.6.0 (675 flowcells) and 4.0.14 (724 flowcells) using the 'hac' model. In v4, all data was basecalled using guppy 5.0.11, using the 'sup' model (dna_r9.4.1_450bps_sup_prom.cfg). All 7,179 individuals basecalled with guppy had a minimum reference-genome-aligned sequencing coverage of at least 10x at the time of analysis. Basecalled reads were mapped to the human reference genome GRCh38 with minimap2, versions 2.14-r883 (5748 flowcells), 2.17-r941 (13 flowcells) and 2.22-r1105 (3145 flowcells). The aligned reads were sorted using samtools sort and stored in a BAM file. Nanopolish v0.11.0 and v0.13.3 were used to detect CpG methylation in nanopore sequences. Data analysis was performed in R (v3.6.0; https://www.r-project.org/).

For manuscripts utilizing custom algorithms or software that are central to the research but not yet described in published literature, software must be made available to editors and reviewers. We strongly encourage code deposition in a community repository (e.g. GitHub). See the Nature Portfolio guidelines for submitting code & software for further information.

Data

Policy information about availability of data

All manuscripts must include a data availability statement. This statement should provide the following information, where applicable:

- Accession codes, unique identifiers, or web links for publicly available datasets
- A description of any restrictions on data availability
- For clinical datasets or third party data, please ensure that the statement adheres to our policy

ASM-QTL summary statistics are available upon request from our website (www.decode.com/summarydata/), and can be used without restrictions. The sequencing data are not publicly available because of Icelandic state law. However, sequence variants identified in the Icelandic population using whole-genome sequencing have been deposited at the European Variant Archive under accession PRJEB15197.

Data from the following publicly available databases were used in the study:
GWAS Catalog: https://www.ebi.ac.uk/gwas/
GTEx project: https://gtexportal.org/home/
eQTLGen: https://www.eqtlgen.org/index.html
Ensembl v.87: https://www.ensembl.org/index.html
VEP: https://www.ensembl.org/info/docs/tools/vep/index.html
NCBI reference genome assembly (hg38; GRCh38): https://www.ncbi.nlm.nih.gov/
ENCODE / Roadmap projects: https://www.encodeproject.org/
Fantom5 project: https://fantom.gsc.riken.jp/5/
AlleleDB: http://alleledb.gersteinlab.org/
LOFTEE: https://github.com/konradjk/loftee

Research involving human participants, their data, or biological material

Policy information about studies with human participants or human data. See also policy information about sex, gender (identity/presentation), and sexual orientation and race, ethnicity and racism.

Reporting on sex and gender	Sex was used as a covariate in our models. Our cohort consisted of 7,179 Icelanders, of which 3,434 were males and 3,745 were females. See Statistics & reproducibility section under methods. Participants were not compensated.
Reporting on race, ethnicity, or other socially relevant groupings	Not applicable.
Population characteristics	A cohort of 7,179 Icelanders (3,434 males, 3,745 females) participating in various studies at deCODE genetics. The earliest year of birth (YOB) was 1876 and 1890 for males and females respectively and the latest was 2015 for both sexes. The median YOB was 1960 for males and 1958 for females. All individuals gave informed consent, and all personal identifiers were encrypted by an external agent before being imported into the deCODE database. See further in methods section "Statistics & reproducibility".
Recruitment	In this study we sequenced DNA isolated from whole blood samples from 7,179 Icelanders participating in various studies at deCODE genetics.
Ethics oversight	The study was approved by the National Bioethics Committee in Iceland (Approval no. VSN 14-015) and conducted in agreement with instructions issued by the Data Protection Authority in Iceland (PV_2017060950ÞS/--).

Note that full information on the approval of the study protocol must also be provided in the manuscript.

Field-specific reporting

Please select the one below that is the best fit for your research. If you are not sure, read the appropriate sections before making your selection.

☒ Life sciences ☐ Behavioural & social sciences ☐ Ecological, evolutionary & environmental sciences

For a reference copy of the document with all sections, see nature.com/documents/nr-reporting-summary-flat.pdf

Life sciences study design

All studies must disclose on these points even when the disclosure is negative.

Sample size	No sample size calculation was performed. The sample size used in the study was determined by the number of nanopore sequenced samples at the time the study was initiated.<br><br>As we detect a large number of statistically significant associations, the sample size is adequate.

Data exclusions	We excluded genomes that were nanopore sequenced to <10x average coverage. We restrict to DNA samples isolated from whole blood samples. These exclusion criterias were pre-established. We restrict to DNA samples isolated from whole blood, thereby excluding DNA isolated from other tissue/cell types.
Replication	The majority (73.4%) of sequence variants identified in association with CpG methylation in an external cohort were replicated in our cohort, see details under Supplementary Note 1.3.<br><br>See also comparison to our oxBS-seq dataset under "Sequence variants influence the 5-mCpG rates of MDSs" in the results section.<br><br>Replication was successful.
Randomization	This is not relevant to our study as we allocate chromosomes (not individuals) into groups based on alleles. We carefully investigated potential confounding variables in our data, see detailed description in the Covariates section under methods.
Blinding	As the study aims to identify methylation-associated sequence variants, blinding was not relevant.

Reporting for specific materials, systems and methods

We require information from authors about some types of materials, experimental systems and methods used in many studies. Here, indicate whether each material, system or method listed is relevant to your study. If you are not sure if a list item applies to your research, read the appropriate section before selecting a response.

Materials & experimental systems

n/a	Involved in the study
☒	☐ Antibodies
☒	☐ Eukaryotic cell lines
☒	☐ Palaeontology and archaeology
☒	☐ Animals and other organisms
☒	☐ Clinical data
☒	☐ Dual use research of concern
☒	☐ Plants

Methods

n/a	Involved in the study
☒	☐ ChIP-seq
☒	☐ Flow cytometry
☒	☐ MRI-based neuroimaging
